# Infectious adverse events associated with immune checkpoint inhibitors: a pharmacovigilance analysis based on FAERS database

**DOI:** 10.3389/fimmu.2025.1647944

**Published:** 2025-10-23

**Authors:** Suting Song, Yana Yang, Qu Hu, Rongjie Zhong, Xuejiao Lei, Chunyu Wang, Ying Wang, Yan Luo

**Affiliations:** ^1^ Radiation Oncology Center, Chongqing University Cancer Hospital, Chongqing, China; ^2^ Health Management Center, The First Affiliated Hospital of Chongqing Medical University, Chongqing, China

**Keywords:** immune checkpoint inhibitors, adverse events, infection, FAERS, immunotherapy

## Abstract

**Background:**

Immune checkpoint inhibitors (ICIs) have revolutionized cancer treatment, but their association with infectious adverse events (iAEs) remains incompletely characterized. These infections may arise from immune dysregulation or immunosuppressive therapies used to manage immune-related toxicities, posing significant clinical challenges. This study aims to define the spectrum, proportion, timing, and clinical outcomes of iAEs in patients treated with ICIs.

**Method:**

Data from the first quarter of 2011 to the fourth quarter of 2023 in FAERS database were extracted to conduct disproportionality analysis. Two signal indices, the reporting odds ratio (ROR) and the information component (IC), which are based on statistical shrinkage transformation, were used to evaluate the correlations between ICIs and immune-related iAEs. Evaluated regimens included ICI monotherapy and combination therapies. Infectious AEs were classified by high-level group terms (HLGTs), high-level terms (HLTs), and preferred terms (PTs) based on the Medical Dictionary for Regulatory Activities (MedDRA), then ranked by frequency and signal strength.

**Results:**

Among 147,854 reports of irAEs, we identified 18068 iAEs demonstrating an overall elevated infection risk (ROR = 1.08, 95% CI [1.07-1.10]) with profound agent-specific heterogeneity. Atezolizumab (ROR = 1.45) and cemiplimab (ROR = 1.42) exhibited the highest risks, while pembrolizumab was associated with a lower risk of iAEs (ROR = 0.82). Disproportionality analyses revealed significant signals for bacterial pneumonia (ROR = 7.49), clostridioides difficile colitis (ROR = 2.11), and pneumocystis jirovecii pneumonia (ROR = 3.78), with pathogen-confirmed cases distributed as bacterial (11.67%), viral (12.20%), and fungal (4.57%) etiologies. Temporal analysis established a critical vulnerability window wherein >70% of iAEs manifested within three months of ICI initiation (median onset 40 days), with pembrolizumab demonstrating the shortest latency (27 days). Age-related disparities revealed that advanced age is associated with increased risk of iAEs following ICI therapy. Combination regimens amplified specific risks, notably encephalitis for nivolumab-ipilimumab (ROR = 17.72), while hospitalization rates reached 71.23% for ipilimumab monotherapy.

**Conclusions:**

This study highlights the significant risk of iAEs in patients treated with ICIs, emphasizing the need for vigilant monitoring, particularly in older patients and those receiving combination therapies. Tailored strategies to prevent and manage infections are essential, and further research is necessary to better understand the mechanisms underlying these adverse events and to refine therapeutic approaches.

## 1 Introduction

Immune checkpoint inhibitors (ICIs) have revolutionized cancer treatment by significantly enhancing antitumor immunity (1). However, their extensive immunomodulatory effects extend beyond antitumor activity, giving rise to a diverse spectrum of immune-related adverse events (irAEs) that increasingly encompass infectious complications. The FDA Adverse Event Reporting System (FAERS) has been effectively utilized to profile infection risks associated with various biologic therapies, including TNF-α inhibitors and interleukin antagonists used for autoimmune conditions (2, 3). However, a comprehensive analysis specifically addressing ICI-associated infections using this large-scale pharmacovigilance database remains lacking. Initially, early clinical trials predominantly characterized irAEs as autoimmune-like toxicities, such as colitis and pneumonitis. Nevertheless, contemporary real-world evidence suggests a notable rise in iAEs associated with ICI utilization (1, 4). This shift underscores the complex interplay between cancer immunotherapy and the immune system, necessitating further research to fully comprehend and manage these associated risks.

The intricate pathophysiologic interplay that exists between ICIs and infections is characterized by two distinct yet interconnected mechanisms. Firstly, the checkpoint blockade, which is a fundamental aspect of ICI therapy, may inadvertently disrupt the delicate balance of immune homeostasis. This disruption can create a permissive environment for opportunistic pathogens to thrive. The paradoxical effect of this immune dysregulation, however, is the hyperactivation of inflammatory pathways, which can lead to a cascade of pathological responses (5). In a cohort study of patients receiving ICIs, bacterial infections were reported in 36.2% (82/226) of cases, while fungal and viral infections occurred in 34.5% (78/226) and 21.2% (48/226), respectively, with polymicrobial infections observed in 8.0% (18/226) of patients (6). In another large retrospective study of non-small cell lung cancer (NSCLC) patients treated with ICIs, 54.4% (162/298) developed infectious complications. Of these patients, 59.3% (96/162) required hospitalization and 15.4% (25/162) required intensive care unit (ICU) admission (37419702). In a study of ICI-treated patients requiring acute hospitalization, 1.2% (18/1561) were admitted to ICU, with immune-mediated toxicities accounting for more than half of these cases, frequently involving infectious complications such as pneumonia (7). iAEs were also correlated with elevated mortality, as evidenced by a fatality rate of 18.33% among reported cases where infection was a contributing factor (8). Moreover, in severe irAEs such as ICI-associated myositis, concurrent infections were identified in 75% of patients and were associated with poor outcomes, including respiratory failure and death (9). Secondly, the immunosuppressive therapies that are often necessary for the management of severe irAEs, such as high-dose corticosteroids, independently predispose patients to a heightened risk of disseminated infections (10). This risk becomes particularly clinically critical when considering that a significant proportion, ranging from 10 to 54.1% of ICI recipients, require prolonged courses of steroid for the treatment of irAE (11, 12).

The current body of evidence concerning the interplay between ICIs, infections, and immune dysregulation remains largely observational in nature. Most of published data has been derived from retrospective case series, which often lack systematically characterization and robust methodologies to establish causality (13–15).It is of critical importance to recognize that the clinical significance of iAEs is exacerbated by the diagnostic and management challenges they present. In contrast to the more commonly understood irAEs, iAEs frequently exhibit symptoms that are similar to or overlap with those of autoimmune toxicity. This overlap can lead to a significant delay in the recognition of these infections and the initiation of appropriate interventions, as evidenced by studies (4, 5). While existing clinical guidelines offer comprehensive protocols for the monitoring of irAEs, they provide only limited guidance on the prevention or mitigation of iAEs. To gain a deeper understanding of the infectious complications associated with ICIs, this particular study undertook a detailed analysis of data sourced from FAERS database. The primary objective of this research is to thoroughly characterize the infection risks, temporal patterns, and outcomes of iAEs that are related to ICIs. This extensive analysis aims to offer additional insights and evidence that will assist healthcare professionals in the clinical application of ICIs, thereby complementing the findings from controlled clinical trials and enhancing patient care.

## 2 Materials and methods

### 2.1 Data source and processing

**Figure 1:**
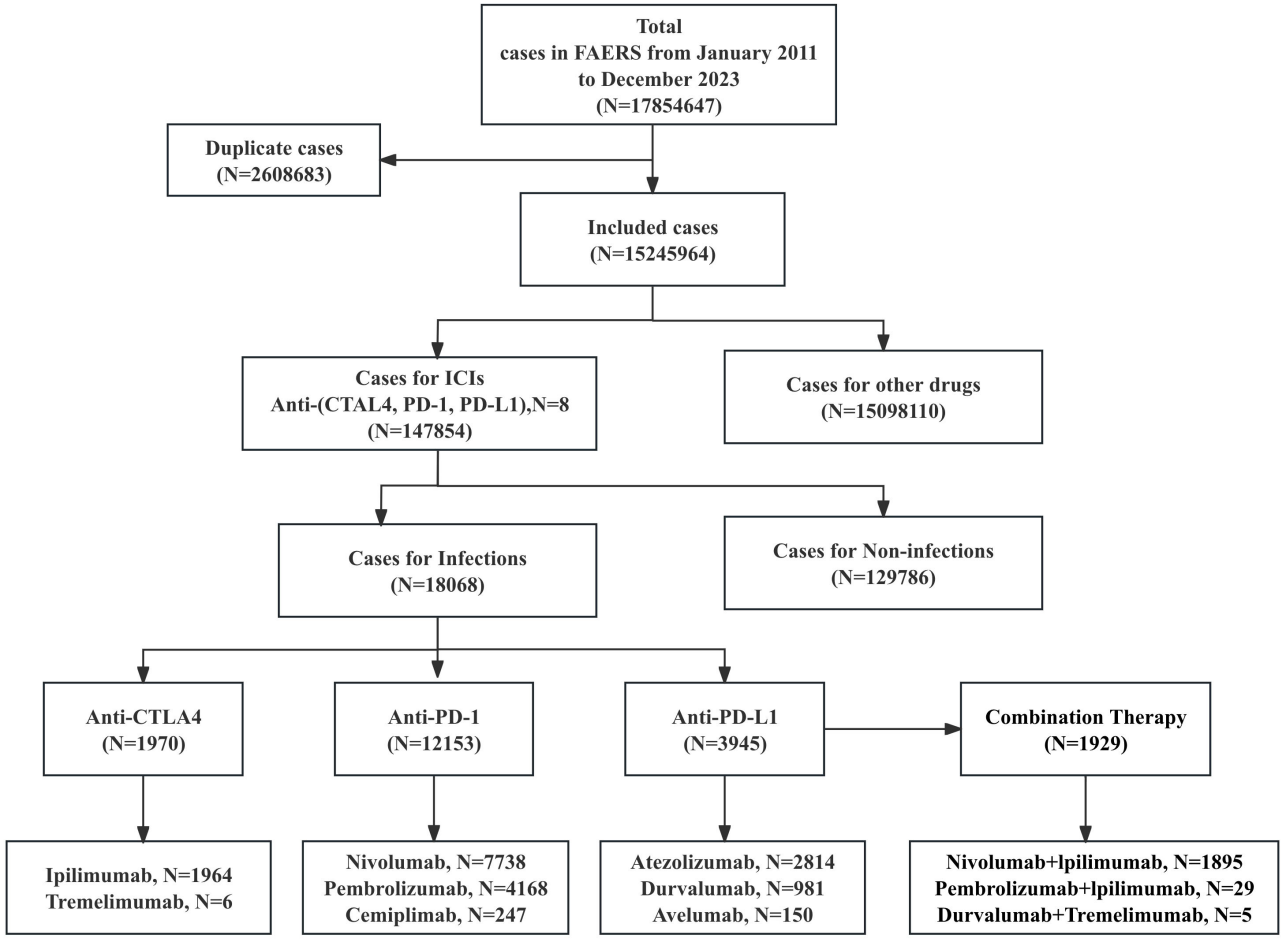
The flow diagram of screening reports from the FAERS database.

This pharmacovigilance study analyzed iAEs associated with ICIs using data from FAERS database (https://open.fda.gov/data/faers/), spanning from the first quarter of 2011 to the fourth quarter of 2023 (**Figure 1**). ICIs of interest included anti-PD-1 (nivolumab, pembrolizumab, cemiplimab), anti-PD-L1 (atezolizumab, avelumab, durvalumab), and anti-CTLA-4 (ipilimumab, tremelimumab) monotherapies and specific combination regimens (nivolumab+ipilimumab, pembrolizumab+ipilimumab, and tremelimumab+durvalumab). Case reports were identified using all relevant drug names, including active ingredients, brand names, and salt forms. Only reports where an ICI was designated as the “Primary Suspect (PS)” drug were included. Combination therapy was defined as the concomitant reporting of two or more different ICI agents (specifically nivolumab+ipilimumab, pembrolizumab+ipilimumab, and tremelimumab+durvalumab), which were analyzed as distinct regimens. Key variables extracted were age, sex, outcomes, drug names, reporting year, country, and event dates. Data cleaning followed FDA recommendations: duplicate reports were removed by retaining the most recent entry based on CASEID and PRIMARYID, prioritizing later FDA_DT and higher PRIMARYID for identical cases. For recurring reports from the same patient, only the latest record (by “FDA data received to date”) was included. This approach prioritizes the minimization of overcounting bias at the potential cost of underestimating the incidence of recurrent adverse events, such as infectious complications. The onset time of iAEs was calculated as the interval between therapy initiation (START_DT) and event onset (EVENT_DT). Reports with invalid dates (START_DT later than EVENT_DT) or missing START_DT/EVENT_DT were excluded. Adverse events were categorized using Medical Dictionary for Regulatory Activities (MedDRA, v26.1) at the System Organ Class (SOC), High-Level Group Terms (HLGTs), High-Level Terms (HLTs), and Preferred Term (PT) levels.

### 2.2 Statistical methods

In pharmacovigilance research, we applied the disproportionality analysis approach to compare the proportion of specific adverse events associated with one or more drugs to the proportion of ADRs for the same drug reported across the entire database. The main specific indicators used to assess drug-related AE signals are reporting odds ratios (ROR) (16)and information components (IC) (17). Statistical analysis methods use a 2 x 2 contingency table to analyze the relationship between a drug and an AE. By calculating the relative frequency of target adverse events in the database over time, these methods evaluate the likelihood of an association. The formula for calculating ROR and IC is as follows:


$$\text{ROR}=\frac{\left(\text{a}/\text{c}\right)}{\left(\text{b}/\text{d}\right)}=\frac{\text{ad}}{\text{bc}}$$

$$\text{IC}={\text{log}}_{2}\frac{\text{a}(\text{a}+\text{b}+\text{c}+\text{d})}{\left(\text{a}+\text{b})(\text{a}+\text{c}\right)}$$

In the formula, ‘a’ represents the number of reports that include both the target drug and its adverse events; ‘b’ represents the number of reports that include adverse events from other drugs along with the target drug; ‘c’ represents the number of reports that include adverse events from the target drug in combination with other drugs; and ‘d’ represents the number of reports that include adverse events from other drugs only. Signal thresholds were defined as: a lower limit of the 95% CI for ROR (ROR_025_) > 1 or a lower limit of the 95% CI for IC (IC_025_) > 0 with at least 3 reports. The primary data management and all statistical analyses, including descriptive statistics and disproportionality analysis, were conducted in SAS version 9.4 (SAS Institute Inc., Cary, NC, United States). Data visualization was performed using specialized software: time-to-onset analyses were plotted with GraphPad Prism 10.0; forest plots were generated using R software (version 4.4.2); and heatmaps were created in Microsoft Excel 2021.

## 3 Results

### 3.1 Basic characteristics of iAEs

This analysis of the FAERS database identified 17,854,647 adverse event reports from the first quarter of 2011 to the fourth quarter of 2023 (**Figure 1**). After deduplication, 15,245,964 cases were analyzed, including 147,854 cases linked to irAEs. Among these, 12.22% (18068/147,854) represented iAEs, which included 1,929 cases derived from combination regimens, while 129,786 cases involved non-infectious irAEs. Demographic analysis (**Table 1**, more details in **Supplementary Tables 1**, **2**) revealed a male predominance (59.84%, N = 10,812) over females (34.13%, N = 6,167), with 1,089 cases (6.03%) lacking gender data. Nearly half of iAEs (48.72%, N = 8,803) occurred in patients aged ≥65 years. Physicians submitted the majority of reports (45.68%), followed by consumers (21.70%), pharmacists (17.20%), and other healthcare professionals (14.42%). Geographically, the United States (33.20%) and Japan (31.09%) accounted for nearly two-thirds of reports, with France (8.97%), Germany (8.40%), and the UK (4.05%) comprising subsequent contributors. Lung cancer was the predominant indication (44.19%, N = 5,926), followed by melanoma (21.61%, N = 2,898) and renal/ureteral malignancies (11.67%, N = 1,565). Hospitalizations represented the most frequent serious outcome (36.50%, N = 12,135), while 21.05% (N = 6,997) involved death or life-threatening events. Anti-PD-1 agents were implicated in 60.78% (N = 12,153) of iAEs, significantly exceeding anti-PD-L1 (19.73%, N = 3,945), anti-CTLA-4 (9.85%, N = 1,970), and combination regimens (9.64%, N = 1,929).

**Table 1:** Clinical characteristics of patients with iAEs.

Characteristics	Infectious AEs of ICIs	Total AEs of ICIs
Gender		
Female	6167 (34.13%)	50135(33.91%)
Male	10812 (59.84%)	80348(54.34%)
Age		
<18	31(0.17%)	340(0.23%)
18-45	911(5.04%)	6959(4.71%)
45-65	5216(28.87%)	37397(25.29%)
≥65	8803(48.72%)	58542(39.59%)
Reporting year		
2011~2018	5455(30.19%)	46977(31.77%)
2019	2349(13.00%)	18248(12.34%)
2020	2278(12.61%)	17787(12.03%)
2021	2433(13.47%)	18992(12.85%)
2022	2667(14.76%)	21689(14.67%)
2023	2886(15.97%)	24161(16.34%)
Reporter type		
Physician	8254(45.68%)	61541(41.62%)
Consumer	3920(21.70%)	38728(26.19%)
Pharmacist	3108(17.20%)	27244(18.42%)
Other health-professional	2605(14.42%)	18886(12.78%)
Reporting countries (Top 5)		
USA	4850(33.20%)	58124(46.56%)
Japan	4542(31.09%)	30953(24.80%)
France	1311(8.97%)	10732(8.60%)
Germany	1228(8.40%)	5810(4.65%)
UK	592(4.05%)	2969(2.38%)
Indication (Top 5)		
Lung Cancer	5926(44.19%)	35620(39.53%)
Malignant Melanoma	2898(21.61%)	18583(20.62%)
Renal and Ureteric Cancer	1565(11.67%)	12131(13.46%)
Hepatobiliary Malignancies	672(5.01%)	6637(7.37%)
Breast Cancer	522(3.89%)	4038(4.48%)
Report type		
Serious	17498(96.85%)	129504(87.59%)
Non-Serious	570(3.15%)	18350(12.41%)
Outcome		
Hospitalization	12135(36.50%)	59077(28.56%)
Death	4906(14.76%)	37726(18.24%)
Life-Threatening	2091(6.29%)	8930(4.32%)
Other Serious	13599(40.91%)	97872(47.32%)

### 3.2 Signal detection related to PT levels

This analysis of pharmacovigilance data identified 18068 cases of iAEs linked to the target drug, part of which were showed in **Table 2** (More details in **Supplementary Table 3**). A notable predominance of cases was categorized as infections-pathogen unspecified (n=15593, 71.55%). Among cases with identified pathogens, viral infections (n=2659, 12.20%), bacterial infections (n=2543, 11.67%), and fungal infections (n=997, 4.57%) were the most frequently reported. Disproportionality signals were assessed using both the ROR and IC. The corresponding IC values and 95% confidence intervals for all reported associations are provided in **Table 2** and **Supplementary Table 3**, and showed high concordance with the ROR-based signals. Disproportionality analysis revealed several strong and significant signals. Pneumonia bacterial demonstrated the highest association among bacterial infections (ROR = 7.49), followed by clostridium difficile colitis (ROR = 2.11). Notable signals were also observed for relapsing fever (ROR = 37.79) and erysipelas (ROR = 2.29). For fungal infections, pneumocystis jirovecii pneumonia was the most significant signal (ROR = 3.78). Among viral infections, coronavirus pneumonia showed a highly elevated signal (ROR = 13.26), whereas COVID-19 and herpes zoster were frequently reported but with RORs below 1. We also identified exceptionally high signals for rare events such as adrenalitis (ROR = 187.80) and enterocolitis infectious (ROR = 10.91). Conversely, bronchitis and influenza showed significant inverse associations (ROR = 0.51 and 0.34, respectively).

**Table 2:** Signal strength of ICI-related iAE at preferred terms (PT).

HLGT	PT	Coding	Cases	ROR(95%CI)	IC (95%CI)
Infections - pathogen unspecified	Pneumonia	10035664	3926	1.81(1.75-1.87)^*^	0.84(0.79-0.89)^*^
	Sepsis	10040047	1737	2.54(2.42-2.66)^*^	1.32(1.25-1.39)^*^
	Urinary tract infection	10046571	1011	0.91(0.86-0.97)	-0.13(-0.22–0.04)
	Encephalitis	10014581	607	17.25(15.83-18.79)^*^	3.91(3.76-4.01)^*^
	Septic shock	10040070	585	2.29(2.11-2.48)^*^	1.18(1.05-1.29)^*^
	Pneumonia aspiration	10035669	568	3.86(3.55-4.2)^*^	1.91(1.78-2.03)^*^
	Nasopharyngitis	10028810	348	0.28(0.25-0.31)	-1.81(-1.96–1.65)
	Bronchitis	10006451	256	0.51(0.45-0.57)	-0.98(-1.15–0.79)
	Meningitis	10027199	241	6.37(5.6-7.26)^*^	2.6(2.38-2.77)^*^
	Meningitis aseptic	10027201	210	8.42(7.32-9.69)^*^	2.98(2.73-3.14)^*^
	Lower respiratory tract infection	10024968	207	0.76(0.66-0.87)	-0.4(-0.6–0.2)
Viral infectious disorders	COVID-19	10084268	752	0.62(0.58-0.66)	-0.69(-0.79–0.58)
	Herpes zoster	10019974	344	0.89(0.8-0.99)	-0.17(-0.33–0.01)
	Influenza	10022000	240	0.34(0.3-0.38)	-1.56(-1.74–1.37)
	COVID-19 pneumonia	10084380	132	1.58(1.33-1.87)^*^	0.65(0.39-0.9)^*^
	Cytomegalovirus infection	10011831	107	1.05(0.87-1.27)	0.07(-0.21-0.34)
	Viral infection	10047461	85	0.42(0.34-0.52)	-1.24(-1.55–0.92)
	Coronavirus infection	10051905	70	1.34(1.06-1.69)^*^	0.42(0.06-0.75)^*^
	Cytomegalovirus enterocolitis	10049015	51	12.6(9.44-16.83)^*^	3.52(2.83-3.67)^*^
Bacterial infectious disorders	Pneumonia bacterial	10060946	381	7.49(6.75-8.31)^*^	2.82(2.65-2.95)^*^
	Cellulitis	10007882	356	1.08(0.97-1.2)	0.11(-0.05-0.26)
	Clostridium difficile colitis	10009657	139	2.11(1.79-2.5)^*^	1.07(0.81-1.3)^*^
	Clostridium difficile infection	10054236	137	0.9(0.76-1.07)	-0.15(-0.39-0.1)
	Staphylococcal infection	10058080	134	0.68(0.57-0.8)	-0.56(-0.81–0.31)
	Bacterial infection	10060945	83	0.76(0.61-0.94)	-0.39(-0.71–0.07)
	Erysipelas	10015145	74	2.29(1.82-2.88)^*^	1.18(0.82-1.49)^*^
	Relapsing fever	10038300	56	37.79(27.92-51.14)^*^	4.84(3.83-4.69)^*^
Fungal infectious disorders	Pneumocystis jirovecii pneumonia	10073755	266	3.78(3.34-4.27)^*^	1.88(1.69-2.05)^*^
	Oral candidiasis	10030963	121	1.61(1.34-1.92)^*^	0.68(0.41-0.93)^*^
	Candida infection	10074170	98	0.78(0.64-0.96)	-0.35(-0.64–0.06)
	Bronchopulmonary aspergillosis	10006473	85	1.83(1.48-2.27)^*^	0.86(0.53-1.16)^*^
	Fungal infection	10017533	76	0.34(0.28-0.43)	-1.53(-1.85–1.19)
	Aspergillus infection	10074171	50	1.08(0.82-1.43)	0.11(-0.29-0.52)
Mycobacterial infectious disorders	Tuberculosis	10044755	77	0.96(0.77-1.2)	-0.06(-0.38-0.27)
	Pulmonary tuberculosis	10037440	75	3.1(2.47-3.9)^*^	1.61(1.23-1.9)^*^
	Atypical mycobacterial infection	10061663	28	3.81(2.62-5.56)^*^	1.9(1.22-2.31)^*^
	Latent tuberculosis	10065048	10	0.71(0.38-1.32)	-0.49(-1.32-0.42)
Ectoparasitic disorders	Acarodermatitis	10063409	6	0.79(0.35-1.75)	-0.35(-1.4-0.79)
	Myiasis	10028586	4	9.8(3.53-27.22)^*^	3.18(0.44-3.14)^*^
Protozoal infectious disorders	Amoebic colitis	10001985	3	5.04(1.59-16.04)^*^	2.28(-0.18-2.78)
	Infection protozoal	10021859	1	4.9(0.66-36.28)	2.24(-1.41-2.83)

Asterisks (*) indicate statistically significant signals in algorithm (ROR_025_ > 1 or IC_025_ > 0, with at least 3 reports); ROR, reporting odds ratio; IC, information components; CI, confidence interval; PT, preferred term; HLGT, high-level group terms.

**Figure 2:**
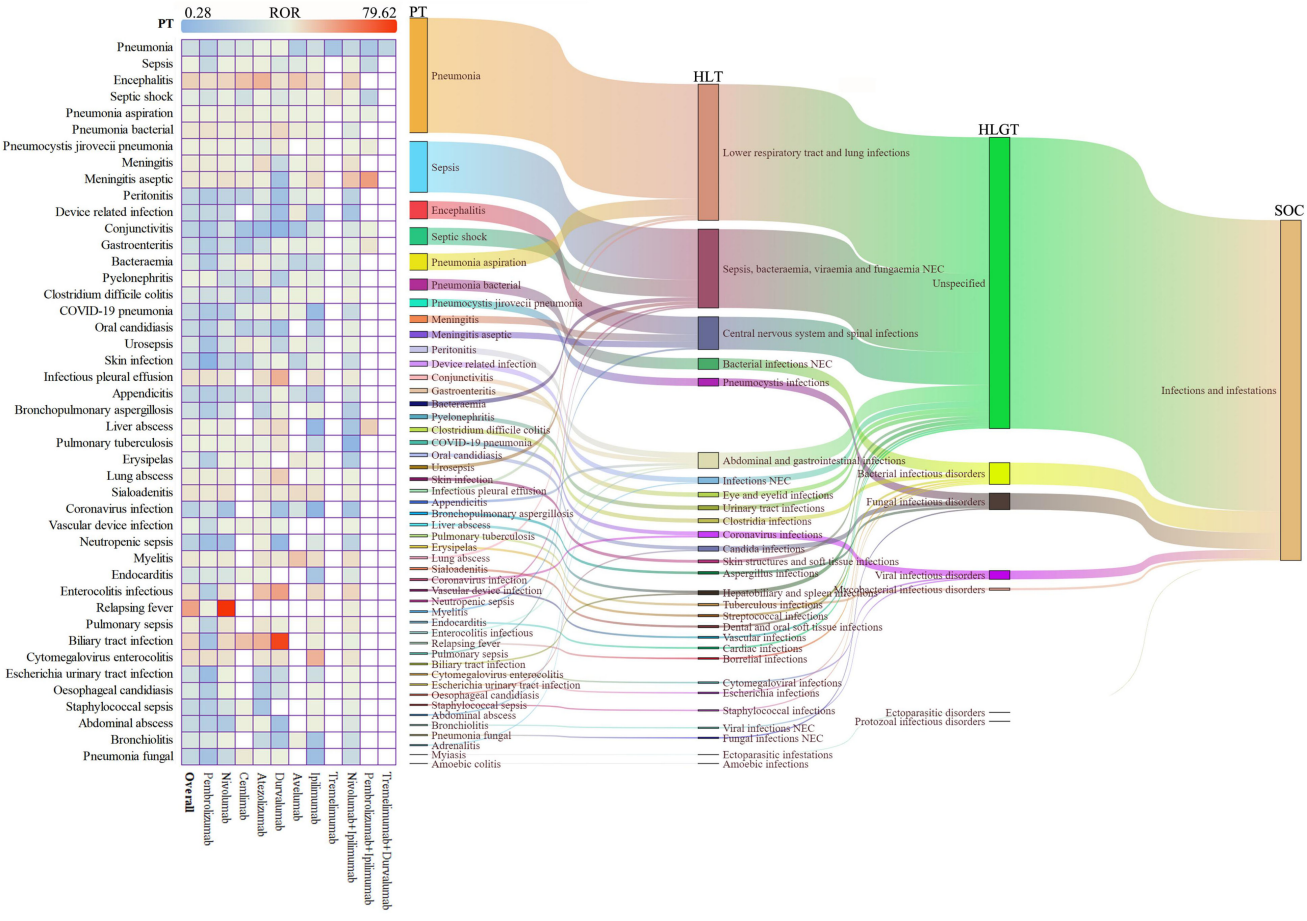
Scanning for ICI-related iAEs based on the FAERS database. The heatmap on the left shows the ROR for iAEs in the FAERS database under different ICI treatment strategies at PTs level. Sankey diagram on the right depicting the hierarchical relationship of PTs for ICI-related iAEs in MedDRA. PT indicates the preferred term, HLT indicates the high-level term, HLGT indicates the high-level group term, and SOC indicates the system organ class.

We conducted systematic visualization of ICI-related iAEs using hierarchical classification from the MedDRA (**Figure 2**, **Supplementary Table 4**). The Sankey diagram illustrated the hierarchical relationship of these infectious adverse events, categorized from broad System Organ Classes (SOCs) like infections and infestations, through more specific High-Level Group Terms (HLGTs) and High-Level Terms (HLTs), down to detailed Preferred Terms (PTs) (Sankey diagrams were generated using the OmicShare tools, https://www.omicshare.com/tools). Categories such as lower respiratory tract infections, lung infections, sepsis, and bacteremia were emphasized, with detailed descriptions of conditions including pneumonia, sepsis, and bacterial infections at the PT level. A heatmap on the left side of **Figure 2** displayed the RORs for different PTs across various ICI regimens (**Figure 2**, **Supplementary Table 5**).

**Figure 3:**
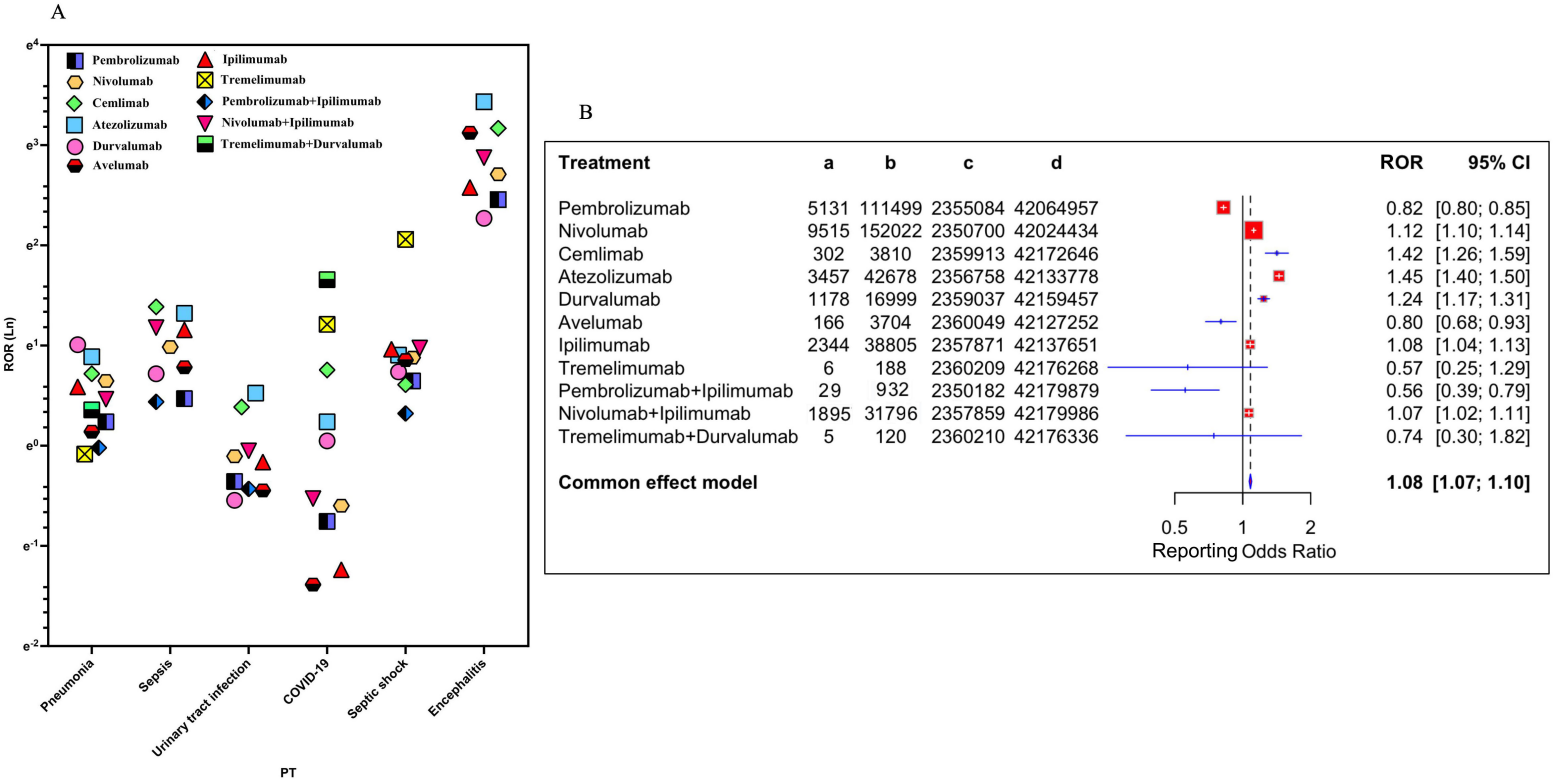
Association of different ICI treatment strategies with ICI−related iAEs. **(A)** The reporting top six ICI-related iAEs at PT level for various treatment strategies were visualized. **(B)** Forest plot shows the reporting odds ratio (ROR) of ICI-related iAEs under different ICI treatment strategies.

Based on the results, the reporting top six ICI-related iAEs at PT level for various treatment strategies were further analyzed (**Figure 3A**, **Supplementary Table 6**). The data showed that encephalitis was the most prominent iAE, with the strongest signals for atezolizumab (ROR = 30.96) and cemiplimab (ROR = 23.76). Sepsis risk was highest for cemiplimab (ROR = 4.00), while septic shock was most significant with nivolumab and atezolizumab. Combination therapies, particularly nivolumab plus ipilimumab, were associated with markedly elevated encephalitis risk (ROR = 17.72). Pneumonia risks were consistently elevated across PD-1/PD-L1 inhibitors (ROR range=1.27-2.74), whereas urinary tract infections (UTI) showed reduced signals for most monotherapies (ROR<1), except atezolizumab (ROR = 1.69). Overall, all ICIs were significantly associated with the increased risk of sepsis, septic shock and encephalitis. A meta-style forest plot of disproportionality across all regimens (**Figure 3B**) confirmed an overall elevated iAE risk (ROR = 1.08, 95% CI [1.07-1.10]). Agent-specific risks varied considerably, with pembrolizumab showing a lower risk (ROR = 0.82) and atezolizumab (ROR = 1.45) and cemiplimab (ROR = 1.42) showing higher risks. Combination treatments such as nivolumab plus ipilimumab demonstrated a modest increase risk of infection (ROR = 1.07).

A comprehensive age-stratified analysis revealed a significantly elevated risk of iAEs in patients aged ≥65 years compared to younger patients, with a pooled ROR of 1.10 (95% CI [1.04–1.16]) across all ICIs (**Supplementary Figure 1**). Moderate heterogeneity (I² = 48.8%) indicated variability in age-related risk among specific agents. Significant increases in iAE reporting were observed with pembrolizumab, nivolumab, and ipilimumab in older patients. In contrast, atezolizumab, durvalumab, and avelumab showed no significant age-dependent risk differences. Cemiplimab suggested a non-significant trend toward lower risk in older patients, while tremelimumab exhibited a large but imprecise effect estimate due to limited data.

### 3.3 Time-to-onset and outcome analysis of iAEs

**Figure 4:**
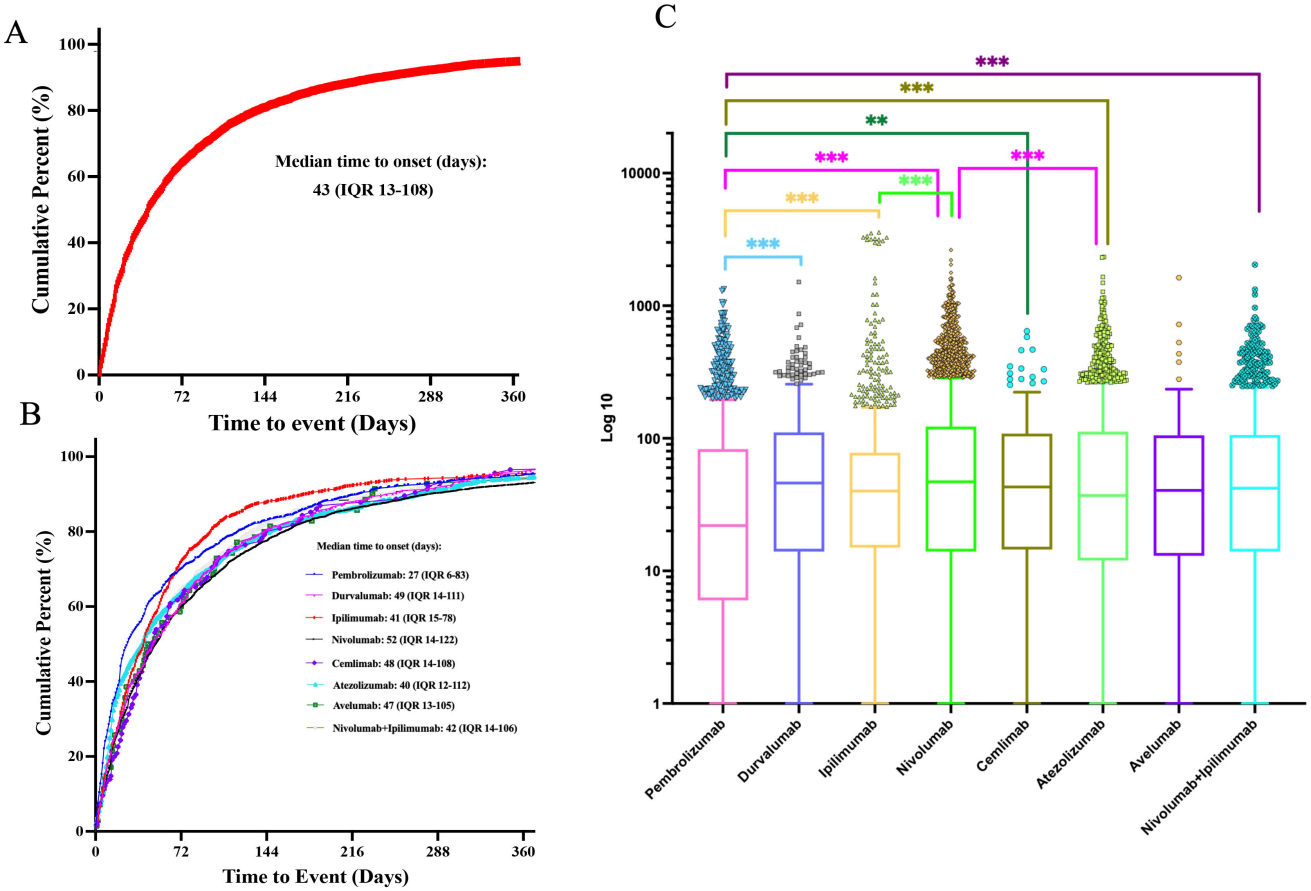
Time-to-onset analysis of ICI-related iAEs. **(A)** The cumulative distribution curves of the onset time of ICI-related iAEs. **(B)** The cumulative distribution curves of the onset time of ICI-related iAEs in different ICI treatment strategies. **(C)** Comparison of onset time of iAEs in various ICI regimens. Statistical tests were conducted using the Kruskal-Wallis’s test. ***P < 0.001.

The time-to-onset analysis included 9,853 iAE cases (54.5% of the total 18,068) with sufficient temporal data after excluding 490 reports with impossible dates. Among these cases, more than 70% of ICI-related iAEs occurred during the first three months after treatment initiation. The median onset time for iAEs was 43 days (Interquartile Range (IQR): 13-108) (**Figures 4A, B**). Importantly, statistical difference in the onset time of iAEs among monotherapy treatments were observed (**Figure 4C**). Notably, pembrolizumab showed the shortest median onset time of 27 days (IQR: 6-83) when compared to other ICI treatment regimens, and this difference was statistically significant (P<0.01). Furthermore, nivolumab achieved the longest median onset time of 52 days (IQR: 14-122), which was significantly longer than that of pembrolizumab, ipilimumab (median 41 days, IQR: 15-78) and cemlimab (median 48 days, IQR: 14-108) (P<0.01). For the combination regimen of nivolumab plus ipilimumab, it achieved median onset time of 42 days (IQR: 14-106).

**Figure 5:**
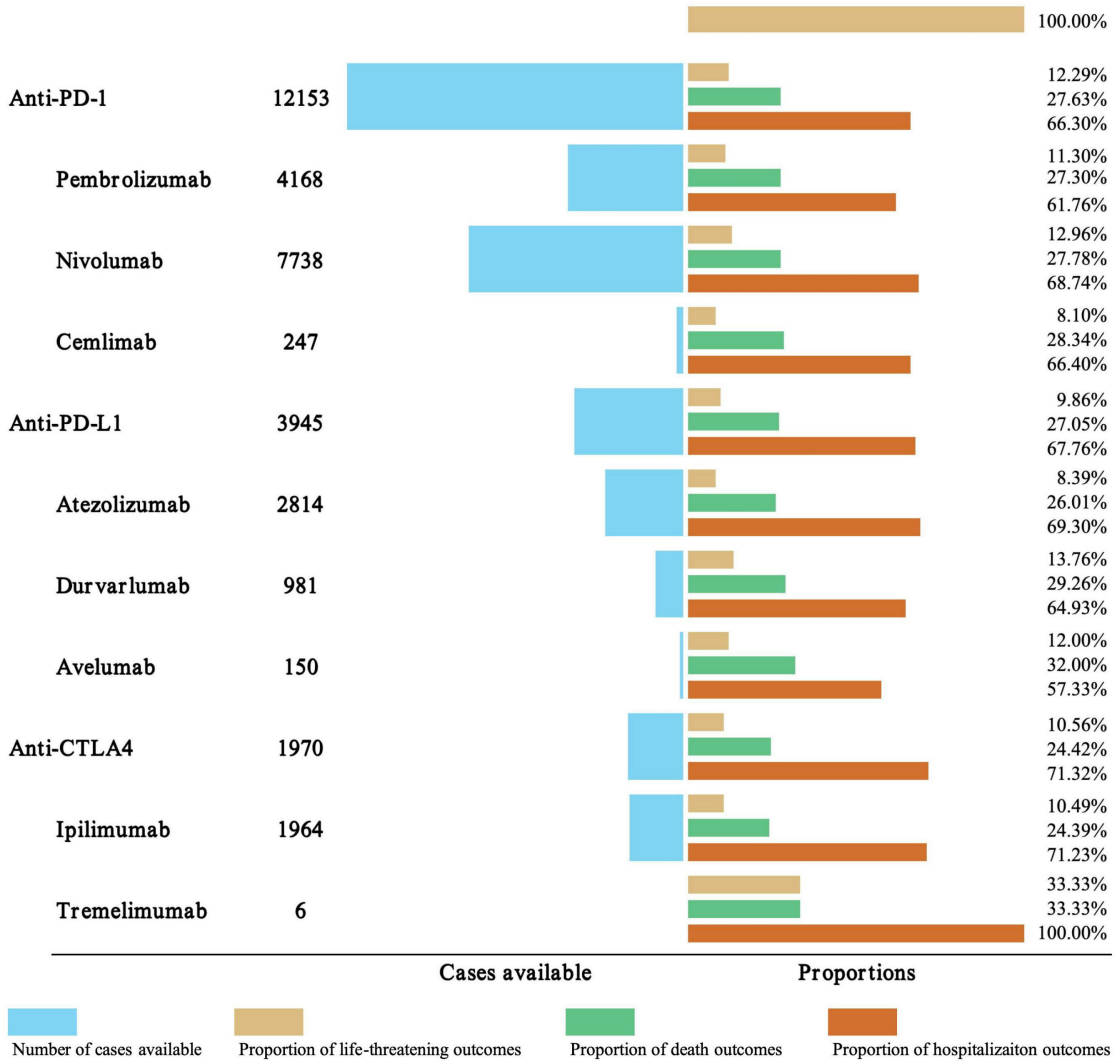
Outcome analysis of ICI-related iAEs. The number of cases, hospitalization, and fatality proportions for ICI-associated iAEs were visualized.

In order to improve the prognosis evaluation of iAEs, we examined the proportions of death, life-threatening, and hospitalization outcomes of different ICI regimen (**Figure 5**). Among anti-PD-1 agents, nivolumab was associated with the highest hospitalization rate (68.74%), followed by pembrolizumab (61.76%) and cemlimab (66.4%), while life-threatening events ranged from 8.1% (cemlimab) to 12.96% (nivolumab). Anti-PD-L1 agents exhibited variability in outcomes. Atezolizumab had the highest hospitalization rate (69.3%), while avelumab had the highest mortality rate (32.00%). This mortality rate for avelumab was notably higher than that of other PD-L1 inhibitors, including durvalumab (29.26%). Anti-CTLA-4 agents displayed marked differences. Ipilimumab accounted for 71.23% of hospitalizations, whereas tremelimumab, despite limited cases (n=6), was associated with disproportionately high rates of life-threatening events (33.33%), mortality (33.33%), and hospitalization (100%). For combination therapies, nivolumab + ipilimumab was associated with a substantially higher hospitalization rate (79.00%) compared to most monotherapies. Its mortality rate (23.01%) was generally comparable to or slightly lower than several PD-1/PD-L1 monotherapies. The limited data for pembrolizumab + ipilimumab (N = 29) and durvalumab + tremelimumab (N = 5) preclude meaningful comparison, though both combinations showed elevated hospitalization and life-threatening event rates.

## 4 Discussion

Our large-scale pharmacovigilance study, analyzing 18,068 iAEs identified from 147,854 irAE reports, confirms that iAEs represent a significant clinical challenge in ICI therapy. While the overall increase in reporting odds (ROR = 1.08) indicates a class-level effect, we observed substantial heterogeneity among individual agents. Atezolizumab (ROR = 1.45) and cemiplimab (ROR = 1.42) showed markedly elevated risks compared to pembrolizumab, suggesting clinically meaningful differences in their safety profiles. This agent-specific risk profile may be explained by distinct pharmacological properties, including variations in Fc-gamma receptor binding affinity that differentially modulate immune cell functions and pathogen surveillance (18). The increased infection risk associated with ICIs generally operates through two interconnected pathways. First, checkpoint blockade directly disrupts immune homeostasis, which can weaken control of opportunistic pathogens (5, 19). Second, the immunosuppressive treatments needed to manage irAEs create additional vulnerability to infections (20).

The clinical impact of this heterogeneity is well illustrated by encephalitis risk patterns. This severe infection showed strong regimen-specific associations, with the highest signal for atezolizumab among monotherapies. This association may reflect viral reactivation or autoimmune-driven neuroinflammation, which has been increasingly reported in real-world cohorts (21, 22). The underrepresentation of such events in RCTs reflects broader limitations in capturing the complete safety profile of ICIs. RCTs typically employ strict inclusion criteria, relatively short follow-up periods, and protocol-directed monitoring that may miss delayed or rare adverse events (23, 24). In contrast, real-world pharmacovigilance studies like ours capture more heterogeneous patient populations and longer-term safety data, often revealing different toxicity patterns than those observed in clinical trials (25, 26). This discrepancy is particularly relevant for infectious complications, which may develop months after treatment initiation and affect patients with comorbidities typically excluded from RCTs (27). Furthermore, combination therapy with nivolumab + ipilimumab substantially amplified this risk beyond single agents, consistent with known toxicity synergism (28, 29). Overall, our analysis demonstrates that infection risk is not uniform across the ICI class but varies substantially by specific agent and treatment strategy. This heterogeneity underscores the importance of regimen-specific vigilance in clinical practice, particularly for high-risk combinations and susceptible patient populations.

The analysis identified elderly patients (≥65 years) and those with lung cancer as subgroups with a higher frequency of reported iAEs. This pattern likely stems from a combination of clinical prescribing trends and biological susceptibility. As lung cancer is a leading indication for ICI therapy and older age is common in treated cancer populations (30, 31), the observed frequencies partly reflect broader treatment patterns. However, the consistent signal in pharmacovigilance data suggests a contribution from biological factors. Age-related immunosenescence can impair pathogen control (32), while lung cancer itself is often associated with compromised respiratory immunity and frequent corticosteroid use, potentially amplifying infection risk. These findings support enhanced vigilance in these patient subgroups. Geographically, the majority of reports originated from the U.S. and Japan, a distribution that likely reflects differences in drug approval, clinical adoption rates, and the maturity of pharmacovigilance systems, rather than implying a true variation in biological risk.

The time-to-onset analysis indicates that over 70% of iAEs occurred within the first three months of treatment, with a median time-to-onset of 40 days. This early risk peak supports current clinical guidelines emphasizing vigilance during initial treatment cycles. However, we observed significant regimen-specific variations in onset kinetics. The markedly shorter latency with pembrolizumab (median 27 days) may reflect its rapid immune activation profile (19, 20), whereas the prolonged interval with nivolumab (52 days) suggests a different immunological dynamic. These distinct timelines indicate that while a general three-month monitoring period is applicable, the peak risk for individual agents may vary. Consequently, monitoring strategies could be optimized by aligning surveillance intensity with these regimen-specific risk periods.

The pathogen-specific analysis provides important insights into the mechanisms underlying ICI-associated infections. The patterns observed suggest that immune checkpoint dysregulation affects host defense through several distinct pathways (19, 33, 34). Bacterial infections, particularly pneumonia and clostridioides difficile colitis, were significantly associated with ICI therapy. This pattern suggests compromised mucosal immunity, a mechanism supported by preclinical studies showing that PD-1 inhibition can alter gut microbiota and impair neutrophil recruitment to infection sites (35). Notably, infections with tuberculosis and herpesviruses were frequently reported in FAERS (**Supplementary Tables 7**, **8**) and corroborated by several reports (36–38). While ICIs can potentially improve control of chronic infections by reversing T-cell exhaustion, they may also precipitate pathological inflammation in cases like cytomegalovirus, where immune hyperactivation exacerbates disease (39, 40). Fungal infections, such as pneumocystis jirovecii pneumonia, represent another important category, indicating that immune dysregulation creates opportunities for opportunistic pathogens. Interestingly, ICIs may also possess potential as antifungal immunotherapies by enhancing protective immune responses, though this application remains investigational (34). A critical finding was the high proportion (71.55%) of infections with unspecified pathogens, underscoring the significant diagnostic challenges in clinical practice. This observation emphasizes the urgent need for improved diagnostic strategies, including advanced molecular techniques, to enable timely and targeted antimicrobial therapy. These pathogen-specific patterns collectively demonstrate that ICI-associated infections arise through diverse mechanisms, necessitating comprehensive diagnostic approaches and tailored management strategies.

Our findings support a risk-stratified approach to the prevention and management of iAEs in patients receiving ICIs. The significant heterogeneity in iAE risk among different agents and regimens necessitates a personalized monitoring strategy. For higher-risk agents, such as atezolizumab and cemiplimab, and for combination therapies like nivolumab + ipilimumab, intensified vigilance is warranted. The observation that over 70% of iAEs occur within the first 90 days of treatment establishes this period as a critical window for patient education and clinical assessment. Monitoring intensity within this window may be further refined based on the distinct onset kinetics of specific agents. For example, earlier and more frequent assessment may be beneficial for patients receiving pembrolizumab given its shorter median time to onset. Older patients (≥65 years), those with lung cancer, and individuals receiving corticosteroids for irAEs represent vulnerable subgroups who may benefit from preemptive evaluation and a low threshold for intervention. The strong signals for specific opportunistic infections, such as bacterial pneumonia, clostridioides difficile colitis, and pneumocystis jirovecii pneumonia (PJP), support the consideration of targeted prophylactic measures in high-risk scenarios. In summary, a proactive management strategy is essential to mitigate the substantial morbidity associated with iAEs while preserving the therapeutic benefits of ICI therapy.

While these analyses provide valuable insights, several limitations warrant careful consideration. As a spontaneous reporting system, the FAERS database is subject to underreporting, selection bias, and variable data quality. Our analytical choices, such as retaining only the most recent report per patient, may further underestimate incidence by excluding recurrent events. The identification of combination therapies from concomitant drug listings could also introduce classification overlap. Importantly, the observational nature of the data precludes causal inference, and confounding by comorbidities or concomitant medications remains possible. Additionally, missing clinical details and lack of standardized follow-up limit comprehensive risk assessment and long-term outcome evaluation. Lastly, inconsistencies in reporting standards and protocols across different countries and healthcare systems result in variations in data completeness and quality, particularly when comparing across regions or regulatory environments. Despite these limitations, the FAERS database provides a unique platform for detecting potential safety signals across large populations. It enables healthcare professionals and researchers to identify potential safety signals and trends. To address the challenges posed by data quality and reporting variability, future studies should focus on using complementary data sources, such as electronic health records or clinical trial data. These will be essential to validate these signals, clarify causal relationships, and establish more precise risk estimates.

## 5 Conclusion

Infections during ICI therapy represent a multifaceted interplay of immune activation, pathogen susceptibility, and iatrogenic immunosuppression. While PD-1/PD-L1 monotherapies offer safer profiles, combination regimens necessitate vigilant risk-benefit evaluations. By exploring real-world data, we advocate for personalized strategies that harmonize oncologic efficacy with infection prevention, ensuring survival gains are not offset by preventable morbidity. As ICIs expand into earlier disease settings, addressing these challenges will be pivotal to optimizing patient outcomes.

## Data Availability

The original contributions presented in the study are included in the article/**Supplementary Material**. Further inquiries can be directed to the corresponding authors.
