# Supplementary material for: Infectious adverse events associated with immune checkpoint inhibitors: a pharmacovigilance analysis based on FAERS database
Source: Front Immunol. 2025 Oct 23;16:1647944. doi: 10.3389/fimmu.2025.1647944 (PMC12589035; doi:10.3389/fimmu.2025.1647944)

***Supplementary Material***

**Supplementary Table 1.** Clinical characteristics of patients with infectious adverse events treated with ICI monotherapy.

|  |  | | |  |
| --- | --- | --- | --- | --- |
|  | Characteristics | Infectious AEs of ICIs | Total AEs of ICIs |  |
|  | Gender |  |  |  |
|  | Female | 6167 (34.13%) | 50135(33.91%) |  |
|  | Male | 10812 (59.84%) | 80348(54.34%) |  |
|  | Not Specified | 1089 (6.03%) | 17371(11.75) |  |
|  | Age |  |  |  |
|  | ＜18 | 31(0.17%) | 340(0.23%) |  |
|  | 18-45 | 911(5.04%) | 6959(4.71%) |  |
|  | 45-65 | 5216(28.87%) | 37397(25.29%) |  |
|  | ≥65 | 8803(48.72%) | 58542(39.59%) |  |
|  | NotSpecified | 3107(17.20%) | 44616(30.18%) |  |
|  | Reporting year |  |  |  |
|  | 2011~2018 | 5455(30.19%) | 46977(31.77%) |  |
|  | 2019 | 2349(13.00%) | 18248(12.34%) |  |
|  | 2020 | 2278(12.61%) | 17787(12.03%) |  |
|  | 2021 | 2433(13.47%) | 18992(12.85%) |  |
|  | 2022 | 2667(14.76%) | 21689(14.67%) |  |
|  | 2023 | 2886(15.97%) | 24161(16.34%) |  |
|  | Reporter Type |  |  |  |
|  | Consumer | 3920(21.70%) | 38728(26.19%) |  |
|  | Lawyer | 9(0.05%) | 55(0.04%) |  |
|  | Not Specified | 172(0.95%) | 1400(0.95%) |  |
|  | Other health-professional | 2605(14.42%) | 18886(12.78%) |  |
|  | Pharmacist | 3108(17.20%) | 27244(18.42%) |  |
|  | Physician | 8254(45.68%) | 61541(41.62%) |  |
|  | Reporting Countries |  |  |  |
|  | USA | 4850(33.20%) | 58124(46.56%) |  |
|  | France | 1311(8.97%) | 10732(8.60%) |  |
|  | Japan | 4542(31.09%) | 30953(24.80%) |  |
|  | Germany | 1228(8.40%) | 5810(4.65%) |  |
|  | Italy | 346(2.37%) | 3109(2.49%) |  |
|  | Spain | 355(2.43%) | 2152(1.72%) |  |
|  | China | 562(3.85%) | 4946(3.96%) |  |
|  | UK | 592(4.05%) | 2969(2.38%) |  |
|  | Australia | 338(2.31%) | 2738(2.19%) |  |
|  | Canada | 486(3.33%) | 3299(2.65%) |  |
|  | Indication |  |  |  |
|  | Lung Cancer | 5926(44.19%) | 35620(39.53%) |  |
|  | Malignant Melanoma | 2898(21.61%) | 18583(20.62%) |  |
|  | Renal and Ureteric Cancer | 1565(11.67%) | 12131(13.46%) |  |
|  | Hepatobiliary Malignancies | 672(5.01%) | 6637(7.37%) |  |
|  | Head and Neck Carcinoma | 254(1.89%) | 1559(1.73%) |  |
|  | Bladder Cancer | 326(2.43%) | 2292(2.54%) |  |
|  | Breast Cancer | 522(3.89%) | 4038(4.48%) |  |
|  | Gastric Cancer | 448(3.34%) | 3617(4.02%) |  |
|  | Colorectal Cancer | 127(0.95%) | 849(0.94%) |  |
|  | Uterine Malignancies | 155(1.15%) | 1247(1.39%) |  |
|  | Oesophageal Cancer | 359(2.68%) | 2523(2.80%) |  |
|  | Prostate Cancer | 159(1.19%) | 1011(1.12%) |  |
|  | Report type |  |  |  |
|  | Serious | 17498(96.85%) | 129504(87.59%) |  |
|  | Non-Serious | 570(3.15%) | 18350(12.41%) |  |
|  | Outcome |  |  |  |
|  | Life-Threatening | 2091(6.29%) | 8930(4.32%) |  |
|  | Hospitalization | 12135(36.50%) | 59077(28.56%) |  |
|  | Disability | 486(1.46%) | 3018(1.46%) |  |
|  | Death | 4906(14.76%) | 37726(18.24%) |  |
|  | Congenital Anomaly | 5(0.01%) | 41(0.02%) |  |
|  | Required Intervention to  Prevent Permanent Impairment | 23(0.07%) | 175(0.08%) |  |
|  | Other Serious | 13599(40.91%) | 97872(47.32%) |  |
|  |  |  |  |  |

**Supplementary Table 2.** Clinical characteristics of patients with infectious adverse events treated with ICI combination therapy.

|  |  |  |  |
| --- | --- | --- | --- |
| Characteristics | Infectious AEs of Nivo+lpi  （n=1895） | Infectious AEs of  Pembro+lpi  （n=29） | Infectious AEs of  Durva+Treme  （n=5） |
| Gender |  |  |  |
| Female | 655(34.57%) | 11(37.93%) | 0(0.00%) |
| Male | 1142(60.26%) | 16(55.17%) | 5(100.00%) |
| Not Specified | 98(5.17%) | 2(6.90%) | 0(0.00%) |
| Age |  |  |  |
| ＜18 | 4(0.21%) | 0(0.00%) | 0(0.00%) |
| 18-45 | 146(7.70%) | 5(17.24%) | 0(0.00%) |
| 45-65 | 670(35.36%) | 11(37.93%) | 1(20.00%) |
| ≥65 | 818(43.17%) | 8(27.59%) | 3(60.00%) |
| NotSpecified | 257(13.56%) | 5(17.24%) | 1(20.00%) |
| Reporting year |  |  |  |
| 2011~2018 | 519(27.39%) | 14(48.28%) | 0(0.00%) |
| 2019 | 313(16.52%) | 1(3.45%) | 0(0.00%) |
| 2020 | 312(16.46%) | 6(20.69%) | 2(40.00%) |
| 2021 | 318(16.78%) | 3(10.34%) | 0(0.00%) |
| 2022 | 311(16.41%) | 1(3.45%) | 0(0.00%) |
| 2023 | 122(6.44%) | 4(13.79%) | 3(60.00%) |
| Reporter Type |  |  |  |
| Consumer | 241(12.72%) | 7(24.14%) | 0(0.00%) |
| Lawyer | 1(0.05%) | 0(0.00%) | 0(0.00%) |
| Not Specified | 4(0.21%) | 1(3.45%) | 0(0.00%) |
| Other health-professional | 536(28.29%) | 5(17.24%) | 0(0.00%) |
| Pharmacist | 581(30.66%) | 12(41.38%) | 1(20.00%) |
| Physician | 532(28.07%) | 4(13.79%) | 4(80.00%) |
| Reporting Countries |  |  |  |
| USA | 671(35.41%) | 15(51.72%) | 0(0.00%) |
| Germany | 259(13.67%) | 3(10.34%) | 0(0.00%) |
| Japan | 195(10.29%) | 0(0.00%) | 4(80.00%) |
| France | 128(6.75%) | 4(13.79%) | 0(0.00%) |
| Canada | 84(4.43%) | 0(0.00%) | 0(0.00%) |
| UK | 77(4.06%) | 1(3.45%) | 0(0.00%) |
| Australia | 53(2.80%) | 1(3.45%) | 0(0.00%) |
| Belgium | 41(2.16%) | 0(0.00%) | 0(0.00%) |
| Spain | 40(2.11%) | 0(0.00%) | 0(0.00%) |
| lreland | 38(2.01%) | 0(0.00%) | 0(0.00%) |
| Indication |  |  |  |
| Malignant Melanoma | 718(37.89%) | 16(55.17%) | 0(0.00%) |
| Lung Cancer | 302(15.94%) | 3(10.34%) | 1(20.00%) |
| Renal and Ureteric Cancer | 360(19.00%) | 0(0.00%) | 0(0.00%) |
| Mesothelioma | 51(2.69%) | 0(0.00%) | 0(0.00%) |
| Colorectal Cancer | 37(1.95%) | 0(0.00%) | 0(0.00%) |
| Transitional cell carcinoma | 27(1.42%) | 0(0.00%) | 1(20.00%) |
| Oesophageal Cancer | 24(1.27%) | 0(0.00%) | 0(0.00%) |
| Prostate Cancer | 18(0.95%) | 0(0.00%) | 0(0.00%) |
| Thyroid cancer | 17(0.90%) | 0(0.00%) | 0(0.00%) |
| Bladder Cancer | 17(0.90%) | 0(0.00%) | 0(0.00%) |
| Report type |  |  |  |
| Serious | 1861(98.21%) | 25(86.21%) | 5(100.00%) |
| Non-Serious | 34(1.79%) | 4(13.79%) | 0(0.00%) |
| Outcome |  |  |  |
| Life-Threatening | 264(13.93%) | 2(6.90%) | 1(20.00%) |
| Hospitalization | 1497(79.00%) | 17(58.62%) | 5(100.00%) |
| Disability | 34(1.79%) | 2(6.90%) | 0(0.00%) |
| Death | 436(23.01%) | 3(10.34%) | 1(20.00%) |
| Congenital Anomaly | 0(0.00%) | 0(0.00%) | 0(0.00%) |
| Required Intervention to  Prevent Permanent Impairment | 0(0.00%) | 0(0.00%) | 0(0.00%) |
| Other Serious | 1661(87.65%) | 21(72.41%) | 3(60.00%) |

Abbreviation: Pembrolizumab, Pembro; Durvalumab, Durva; Nivolumab, Nivo; Tremelimumab, Treme; lpilimumab, Ipi.

**Supplementary Table 3.** Signal strength of immune-related infectious adverse events at Preferred Terms (PT).

| **HLGT** | **PT** | **Coding** | **Cases** | **ROR（95%CI）** | **IC(95%CI）** |
| --- | --- | --- | --- | --- | --- |
| Infections - pathogen unspecified | Pneumonia | 10035664 | 3926 | 1.81(1.75-1.87)* | 0.84(0.79-0.89)* |
|  | Sepsis | 10040047 | 1737 | 2.54(2.42-2.66)* | 1.32(1.25-1.39)* |
|  | Urinary tract infection | 10046571 | 1011 | 0.91(0.86-0.97) | -0.13(-0.22--0.04) |
|  | Infection | 10021789 | 765 | 0.84(0.78-0.9) | -0.26(-0.36--0.15) |
|  | Encephalitis | 10014581 | 607 | 17.25(15.83-18.79)* | 3.91(3.76-4.01)* |
|  | Septic shock | 10040070 | 585 | 2.29(2.11-2.48)* | 1.18(1.05-1.29)* |
|  | Pneumonia aspiration | 10035669 | 568 | 3.86(3.55-4.2)* | 1.91(1.78-2.03)* |
|  | Nasopharyngitis | 10028810 | 348 | 0.28(0.25-0.31) | -1.81(-1.96--1.65) |
|  | Bronchitis | 10006451 | 256 | 0.51(0.45-0.57) | -0.98(-1.15--0.79) |
|  | Meningitis | 10027199 | 241 | 6.37(5.6-7.26)* | 2.6(2.38-2.77)* |
|  | Meningitis aseptic | 10027201 | 210 | 8.42(7.32-9.69)* | 2.98(2.73-3.14)* |
|  | Lower respiratory tract infection | 10024968 | 207 | 0.76(0.66-0.87) | -0.4(-0.6--0.2) |
|  | Peritonitis | 10034674 | 206 | 1.5(1.31-1.72)* | 0.58(0.37-0.78)* |
|  | Diverticulitis | 10013538 | 193 | 1.09(0.95-1.26) | 0.12(-0.09-0.33) |
|  | Device related infection | 10064687 | 175 | 1.57(1.36-1.83)* | 0.65(0.42-0.86)* |
|  | Respiratory tract infection | 10062352 | 157 | 0.97(0.83-1.13) | -0.05(-0.28-0.18) |
|  | Conjunctivitis | 10010741 | 154 | 1.38(1.18-1.62)* | 0.46(0.22-0.69)* |
|  | Gastroenteritis | 10017888 | 152 | 1.75(1.5-2.06)* | 0.8(0.56-1.03)* |
|  | Cystitis | 10011781 | 149 | 0.69(0.59-0.81) | -0.54(-0.77--0.3) |
|  | Upper respiratory tract infection | 10046306 | 145 | 0.49(0.41-0.57) | -1.04(-1.27--0.79) |
|  | Sinusitis | 10040753 | 145 | 0.21(0.18-0.25) | -2.22(-2.45--1.97) |
|  | Bacteraemia | 10003997 | 143 | 2.03(1.72-2.4)* | 1.01(0.76-1.24)* |
|  | Pyelonephritis | 10037596 | 142 | 2.7(2.29-3.19)* | 1.41(1.15-1.64)* |
|  | Urosepsis | 10048709 | 115 | 1.99(1.65-2.39)* | 0.98(0.7-1.24)* |
|  | Skin infection | 10040872 | 98 | 1.36(1.11-1.65)* | 0.43(0.14-0.72)* |
|  | Infectious pleural effusion | 10071699 | 92 | 10.16(8.21-12.58)* | 3.23(2.8-3.42)* |
|  | Appendicitis | 10003011 | 85 | 1.52(1.23-1.88)* | 0.6(0.28-0.9)* |
|  | Liver abscess | 10024652 | 84 | 4.37(3.51-5.43)* | 2.09(1.71-2.35)* |
|  | Lung abscess | 10025028 | 73 | 6.05(4.78-7.66)* | 2.53(2.1-2.79)* |
|  | Wound infection | 10048038 | 72 | 1.23(0.97-1.55) | 0.29(-0.05-0.63) |
| Viral infectious disorders | COVID-19 | 10084268 | 752 | 0.62(0.58-0.66) | -0.69(-0.79--0.58) |
|  | Herpes zoster | 10019974 | 344 | 0.89(0.8-0.99) | -0.17(-0.33--0.01) |
|  | Influenza | 10022000 | 240 | 0.34(0.3-0.38) | -1.56(-1.74--1.37) |
|  | COVID-19 pneumonia | 10084380 | 132 | 1.58(1.33-1.87)* | 0.65(0.39-0.9)* |
|  | Cytomegalovirus infection | 10011831 | 107 | 1.05(0.87-1.27) | 0.07(-0.21-0.34) |
|  | Viral infection | 10047461 | 85 | 0.42(0.34-0.52) | -1.24(-1.55--0.92) |
|  | Coronavirus infection | 10051905 | 70 | 1.34(1.06-1.69)* | 0.42(0.06-0.75)* |
|  | Cytomegalovirus enterocolitis | 10049015 | 51 | 12.6(9.44-16.83)* | 3.52(2.83-3.67)* |
|  | Oral herpes | 10067152 | 49 | 0.39(0.3-0.52) | -1.35(-1.74--0.92) |
|  | Bronchiolitis | 10006448 | 43 | 2.02(1.5-2.74)* | 1(0.53-1.41)* |
|  | Cytomegalovirus colitis | 10048983 | 35 | 2.73(1.95-3.81)* | 1.42(0.87-1.84)* |
|  | Hepatitis C | 10019744 | 35 | 0.41(0.29-0.57) | -1.28(-1.73--0.77) |
|  | Hepatitis B reactivation | 10058827 | 32 | 1.44(1.01-2.04)* | 0.52(-0.01-1) |
|  | Gastroenteritis viral | 10017918 | 31 | 0.27(0.19-0.38) | -1.89(-2.37--1.35) |
|  | Herpes virus infection | 10019973 | 30 | 0.85(0.59-1.22) | -0.23(-0.74-0.3) |
|  | Progressive multifocal leukoencephalopathy | 10036807 | 29 | 0.54(0.37-0.77) | -0.89(-1.4--0.34) |
|  | Pneumonia viral | 10035737 | 28 | 1.45(1-2.11)* | 0.53(-0.03-1.05) |
|  | Hepatitis B | 10019731 | 26 | 0.72(0.49-1.06) | -0.47(-1.01-0.1) |
|  | Herpes simplex | 10019948 | 22 | 0.74(0.49-1.13) | -0.42(-1.01-0.2) |
|  | Pneumonia cytomegaloviral | 10035676 | 20 | 1.79(1.15-2.78)* | 0.83(0.14-1.41)* |
|  | Epstein-Barr virus infection | 10015108 | 20 | 0.54(0.35-0.84) | -0.88(-1.48--0.22) |
|  | Suspected COVID-19 | 10084451 | 19 | 0.71(0.46-1.12) | -0.48(-1.11-0.19) |
|  | Hepatitis E | 10019768 | 16 | 1.49(0.91-2.43) | 0.57(-0.18-1.23) |
|  | Meningitis viral | 10027260 | 15 | 1.86(1.12-3.1)* | 0.89(0.08-1.54)* |
|  | Meningoencephalitis herpetic | 10027285 | 14 | 3.24(1.9-5.51)* | 1.67(0.71-2.23)* |
|  | Viral upper respiratory tract infection | 10047482 | 14 | 0.38(0.23-0.65) | -1.37(-2.06--0.57) |
|  | Rhinovirus infection | 10061494 | 13 | 0.6(0.35-1.03) | -0.74(-1.47-0.08) |
|  | Cytomegalovirus viraemia | 10058854 | 13 | 0.51(0.3-0.88) | -0.96(-1.68--0.13) |
|  | Vestibular neuronitis | 10047393 | 13 | 4.2(2.41-7.3)* | 2.03(0.95-2.53)* |
|  | Hepatitis viral | 10019799 | 12 | 3.31(1.86-5.87)* | 1.7(0.65-2.28)* |
| Bacterial infectious disorders | Pneumonia bacterial | 10060946 | 381 | 7.49(6.75-8.31)* | 2.82(2.65-2.95)* |
|  | Cellulitis | 10007882 | 356 | 1.08(0.97-1.2) | 0.11(-0.05-0.26) |
|  | Clostridium difficile colitis | 10009657 | 139 | 2.11(1.79-2.5)* | 1.07(0.81-1.3)* |
|  | Clostridium difficile infection | 10054236 | 137 | 0.9(0.76-1.07) | -0.15(-0.39-0.1) |
|  | Staphylococcal infection | 10058080 | 134 | 0.68(0.57-0.8) | -0.56(-0.81--0.31) |
|  | Bacterial infection | 10060945 | 83 | 0.76(0.61-0.94) | -0.39(-0.71--0.07) |
|  | Erysipelas | 10015145 | 74 | 2.29(1.82-2.88)* | 1.18(0.82-1.49)* |
|  | Relapsing fever | 10038300 | 56 | 37.79(27.92-51.14)* | 4.84(3.83-4.69)* |
|  | Escherichia urinary tract infection | 10052238 | 48 | 1.82(1.37-2.42)* | 0.86(0.42-1.25)* |
|  | Escherichia infection | 10061126 | 46 | 1.01(0.75-1.34) | 0.01(-0.42-0.43) |
|  | Staphylococcal sepsis | 10056430 | 44 | 1.59(1.18-2.14)* | 0.66(0.21-1.08)* |
|  | Pseudomembranous colitis | 10037128 | 38 | 3.05(2.21-4.21)* | 1.58(1.04-1.98)* |
|  | Pseudomonas infection | 10061471 | 37 | 0.79(0.57-1.09) | -0.34(-0.8-0.14) |
|  | Escherichia sepsis | 10015296 | 36 | 2.05(1.47-2.85)* | 1.02(0.5-1.46)* |
|  | Paronychia | 10034016 | 35 | 1.27(0.91-1.78) | 0.34(-0.15-0.82) |
|  | Pneumonia klebsiella | 10035717 | 31 | 2.91(2.04-4.16)* | 1.52(0.92-1.95)* |
|  | Staphylococcal bacteraemia | 10051017 | 29 | 1.21(0.84-1.74) | 0.27(-0.27-0.79) |
|  | Urinary tract infection bacterial | 10054088 | 29 | 1.7(1.18-2.45)* | 0.76(0.19-1.25)* |
|  | Pneumonia staphylococcal | 10035734 | 25 | 2.38(1.6-3.53)* | 1.23(0.58-1.73)* |
|  | Bacterial sepsis | 10053840 | 25 | 1.34(0.9-1.99) | 0.42(-0.17-0.97) |
|  | Folliculitis | 10016936 | 23 | 0.5(0.33-0.76) | -0.98(-1.54--0.36) |
|  | Furuncle | 10017553 | 23 | 0.43(0.28-0.64) | -1.22(-1.78--0.6) |
|  | Pneumonia pneumococcal | 10035728 | 21 | 2.55(1.66-3.93)* | 1.33(0.61-1.86)* |
|  | Escherichia bacteraemia | 10054258 | 20 | 1.53(0.98-2.38) | 0.61(-0.06-1.2) |
|  | Klebsiella infection | 10061259 | 20 | 0.7(0.45-1.09) | -0.51(-1.12-0.14) |
|  | Peritonitis bacterial | 10062070 | 19 | 0.29(0.19-0.46) | -1.76(-2.35--1.06) |
|  | Urinary tract infection enterococcal | 10046572 | 19 | 3.43(2.17-5.41)* | 1.75(0.93-2.24)* |
|  | Pneumonia pseudomonal | 10035731 | 18 | 1.53(0.96-2.44) | 0.61(-0.1-1.24) |
|  | Gangrene | 10017711 | 18 | 0.45(0.28-0.72) | -1.14(-1.76--0.44) |
|  | Enterococcal infection | 10061124 | 17 | 0.67(0.41-1.07) | -0.58(-1.24-0.13) |
| Fungal infectious disorders | Pneumocystis jirovecii pneumonia | 10073755 | 266 | 3.78(3.34-4.27)* | 1.88(1.69-2.05)* |
|  | Oral candidiasis | 10030963 | 121 | 1.61(1.34-1.92)* | 0.68(0.41-0.93)* |
|  | Candida infection | 10074170 | 98 | 0.78(0.64-0.96) | -0.35(-0.64--0.06) |
|  | Bronchopulmonary aspergillosis | 10006473 | 85 | 1.83(1.48-2.27)* | 0.86(0.53-1.16)* |
|  | Fungal infection | 10017533 | 76 | 0.34(0.28-0.43) | -1.53(-1.85--1.19) |
|  | Aspergillus infection | 10074171 | 50 | 1.08(0.82-1.43) | 0.11(-0.29-0.52) |
|  | Oesophageal candidiasis | 10030154 | 46 | 2.04(1.53-2.73)* | 1.02(0.56-1.41)* |
|  | Pneumonia fungal | 10061354 | 43 | 1.55(1.15-2.1)* | 0.63(0.17-1.05)* |
|  | Oral fungal infection | 10061324 | 27 | 1.6(1.09-2.33)* | 0.67(0.09-1.19)* |
|  | Systemic candida | 10042938 | 20 | 1.41(0.9-2.18) | 0.49(-0.18-1.09) |
| Mycobacterial infectious disorders | Tuberculosis | 10044755 | 77 | 0.96(0.77-1.2) | -0.06(-0.38-0.27) |
|  | Pulmonary tuberculosis | 10037440 | 75 | 3.1(2.47-3.9)* | 1.61(1.23-1.9)* |
|  | Atypical mycobacterial infection | 10061663 | 28 | 3.81(2.62-5.56)* | 1.9(1.22-2.31)* |
|  | Latent tuberculosis | 10065048 | 10 | 0.71(0.38-1.32) | -0.49(-1.32-0.42) |
|  | Mycobacterial infection | 10062207 | 9 | 0.82(0.43-1.59) | -0.28(-1.17-0.66) |
|  | Mycobacterium avium complex infection | 10058806 | 9 | 0.63(0.33-1.22) | -0.66(-1.52-0.31) |
|  | Tuberculous pleurisy | 10045104 | 7 | 2.49(1.18-5.26)* | 1.3(0.02-2.08)* |
|  | Lymph node tuberculosis | 10025183 | 6 | 1.31(0.58-2.92) | 0.38(-0.78-1.41) |
|  | Disseminated tuberculosis | 10013453 | 6 | 0.45(0.2-1) | -1.16(-2.13-0.06) |
|  | Disseminated Bacillus Calmette-Guerin infection | 10076666 | 5 | 1.48(0.61-3.57) | 0.56(-0.74-1.63) |
| Ectoparasitic disorders | Acarodermatitis | 10063409 | 6 | 0.79(0.35-1.75) | -0.35(-1.4-0.79) |
|  | Myiasis | 10028586 | 4 | 9.8(3.53-27.22)* | 3.18(0.44-3.14)* |
|  | Infestation | 10061217 | 2 | 1.21(0.3-4.88) | 0.27(-1.51-1.85) |
| Protozoal infectious disorders | Amoebic colitis | 10001985 | 3 | 5.04(1.59-16.04)* | 2.28(-0.18-2.78) |
|  | Infection protozoal | 10021859 | 1 | 4.9(0.66-36.28) | 2.24(-1.41-2.83) |
|  | Giardiasis | 10018262 | 1 | 0.53(0.07-3.81) | -0.9(-2.57-1.53) |
| Ancillary infectious topics | Nosocomial infection | 10029803 | 6 | 0.42(0.19-0.94) | -1.25(-2.21--0.03) |
|  | Iatrogenic infection | 10064091 | 1 | 2.3(0.32-16.65) | 1.18(-1.61-2.55) |
|  | Bacterial disease carrier | 10004017 | 1 | 0.2(0.03-1.42) | -2.32(-3.63-0.46) |
| Helminthic disorders | Toxocariasis | 10044269 | 2 | 10.24(2.41-43.56) | 3.24(-0.46-3.06) |
|  | Helminthic infection | 10061201 | 2 | 1.17(0.29-4.73) | 0.23(-1.53-1.82) |
|  | Strongyloidiasis | 10042254 | 1 | 0.1(0.01-0.69) | -3.35(-4.53--0.44) |
| Chlamydial infectious disorders | Chlamydial infection | 10061041 | 5 | 1.01(0.42-2.44) | 0.02(-1.17-1.2) |
|  | Pneumonia chlamydial | 10035673 | 2 | 2.71(0.67-11.04) | 1.42(-0.92-2.47) |
| Mycoplasmal infectious disorders | Pneumonia mycoplasmal | 10035724 | 5 | 1.09(0.45-2.64) | 0.13(-1.08-1.29) |
|  | Mycoplasma infection | 10061300 | 1 | 0.29(0.04-2.05) | -1.79(-3.2-0.89) |
| Rickettsial infectious disorders | Q fever | 10037688 | 3 | 1.99(0.63-6.23) | 0.98(-0.8-2.12) |
|  | Rocky mountain spotted fever | 10039207 | 2 | 1.39(0.34-5.61) | 0.47(-1.39-1.97) |

Asterisks (*) indicate statistically significant signals in algorithm; ROR, reporting odds ratio; IC, information components; CI, confidence interval; PT, preferred term; HLGT, high-level group terms.

**Supplementary Table 4.** Data for Sankey visualization

| **PT** | **HLT** | **Cases** |
| --- | --- | --- |
| Pneumonia | Lower respiratory tract and lung infections | 3926 |
| Sepsis | Sepsis, bacteraemia, viraemia and fungaemia NEC | 1737 |
| Encephalitis | Central nervous system and spinal infections | 607 |
| Septic shock | Sepsis, bacteraemia, viraemia and fungaemia NEC | 585 |
| Pneumonia aspiration | Lower respiratory tract and lung infections | 568 |
| Pneumonia bacterial | Bacterial infections NEC | 381 |
| Pneumocystis jirovecii pneumonia | Pneumocystis infections | 266 |
| Meningitis | Central nervous system and spinal infections | 241 |
| Meningitis aseptic | Central nervous system and spinal infections | 210 |
| Peritonitis | Abdominal and gastrointestinal infections | 206 |
| Device related infection | Infections NEC | 175 |
| Conjunctivitis | Eye and eyelid infections | 154 |
| Gastroenteritis | Abdominal and gastrointestinal infections | 152 |
| Bacteraemia | Sepsis, bacteraemia, viraemia and fungaemia NEC | 143 |
| Pyelonephritis | Urinary tract infections | 142 |
| Clostridium difficile colitis | Clostridia infections | 139 |
| COVID-19 pneumonia | Coronavirus infections | 132 |
| Oral candidiasis | Candida infections | 121 |
| Urosepsis | Sepsis, bacteraemia, viraemia and fungaemia NEC | 115 |
| Skin infection | Skin structures and soft tissue infections | 98 |
| Infectious pleural effusion | Lower respiratory tract and lung infections | 92 |
| Appendicitis | Abdominal and gastrointestinal infections | 85 |
| Bronchopulmonary aspergillosis | Aspergillus infections | 85 |
| Liver abscess | Hepatobiliary and spleen infections | 84 |
| Pulmonary tuberculosis | Tuberculous infections | 75 |
| Erysipelas | Streptococcal infections | 74 |
| Lung abscess | Lower respiratory tract and lung infections | 73 |
| Sialoadenitis | Dental and oral soft tissue infections | 71 |
| Coronavirus infection | Coronavirus infections | 70 |
| Vascular device infection | Vascular infections | 63 |
| Neutropenic sepsis | Sepsis, bacteraemia, viraemia and fungaemia NEC | 62 |
| Myelitis | Central nervous system and spinal infections | 61 |
| Endocarditis | Cardiac infections | 60 |
| Enterocolitis infectious | Abdominal and gastrointestinal infections | 57 |
| Relapsing fever | Borrelial infections | 56 |
| Pulmonary sepsis | Sepsis, bacteraemia, viraemia and fungaemia NEC | 55 |
| Biliary tract infection | Hepatobiliary and spleen infections | 54 |
| Cytomegalovirus enterocolitis | Cytomegaloviral infections | 51 |
| Escherichia urinary tract infection | Escherichia infections | 48 |
| Oesophageal candidiasis | Candida infections | 46 |
| Staphylococcal sepsis | Staphylococcal infections | 44 |
| Abdominal abscess | Abdominal and gastrointestinal infections | 44 |
| Bronchiolitis | Viral infections NEC | 43 |
| Pneumonia fungal | Fungal infections NEC | 43 |
| Adrenalitis | Infections NEC | 40 |
| Myiasis | Ectoparasitic infestations | 4 |
| Amoebic colitis | Amoebic infections | 3 |
| **HLT** | **HLGT** | **Cases** |
| Lower respiratory tract and lung infections | Infections - pathogen unspecified | 3926 |
| Sepsis, bacteraemia, viraemia and fungaemia NEC | Infections - pathogen unspecified | 1737 |
| Central nervous system and spinal infections | Infections - pathogen unspecified | 607 |
| Sepsis, bacteraemia, viraemia and fungaemia NEC | Infections - pathogen unspecified | 585 |
| Lower respiratory tract and lung infections | Infections - pathogen unspecified | 568 |
| Bacterial infections NEC | Bacterial infectious disorders | 381 |
| Pneumocystis infections | Fungal infectious disorders | 266 |
| Central nervous system and spinal infections | Infections - pathogen unspecified | 241 |
| Central nervous system and spinal infections | Infections - pathogen unspecified | 210 |
| Abdominal and gastrointestinal infections | Infections - pathogen unspecified | 206 |
| Infections NEC | Infections - pathogen unspecified | 175 |
| Eye and eyelid infections | Infections - pathogen unspecified | 154 |
| Abdominal and gastrointestinal infections | Infections - pathogen unspecified | 152 |
| Sepsis, bacteraemia, viraemia and fungaemia NEC | Infections - pathogen unspecified | 143 |
| Urinary tract infections | Infections - pathogen unspecified | 142 |
| Clostridia infections | Bacterial infectious disorders | 139 |
| Coronavirus infections | Viral infectious disorders | 132 |
| Candida infections | Fungal infectious disorders | 121 |
| Sepsis, bacteraemia, viraemia and fungaemia NEC | Infections - pathogen unspecified | 115 |
| Skin structures and soft tissue infections | Infections - pathogen unspecified | 98 |
| Lower respiratory tract and lung infections | Infections - pathogen unspecified | 92 |
| Abdominal and gastrointestinal infections | Infections - pathogen unspecified | 85 |
| Aspergillus infections | Fungal infectious disorders | 85 |
| Hepatobiliary and spleen infections | Infections - pathogen unspecified | 84 |
| Tuberculous infections | Mycobacterial infectious disorders | 75 |
| Streptococcal infections | Bacterial infectious disorders | 74 |
| Lower respiratory tract and lung infections | Infections - pathogen unspecified | 73 |
| Dental and oral soft tissue infections | Infections - pathogen unspecified | 71 |
| Coronavirus infections | Viral infectious disorders | 70 |
| Vascular infections | Infections - pathogen unspecified | 63 |
| Sepsis, bacteraemia, viraemia and fungaemia NEC | Infections - pathogen unspecified | 62 |
| Central nervous system and spinal infections | Infections - pathogen unspecified | 61 |
| Cardiac infections | Infections - pathogen unspecified | 60 |
| Abdominal and gastrointestinal infections | Infections - pathogen unspecified | 57 |
| Borrelial infections | Bacterial infectious disorders | 56 |
| Sepsis, bacteraemia, viraemia and fungaemia NEC | Infections - pathogen unspecified | 55 |
| Hepatobiliary and spleen infections | Infections - pathogen unspecified | 54 |
| Cytomegaloviral infections | Viral infectious disorders | 51 |
| Escherichia infections | Bacterial infectious disorders | 48 |
| Candida infections | Fungal infectious disorders | 46 |
| Staphylococcal infections | Bacterial infectious disorders | 44 |
| Abdominal and gastrointestinal infections | Infections - pathogen unspecified | 44 |
| Viral infections NEC | Viral infectious disorders | 43 |
| Fungal infections NEC | Fungal infectious disorders | 43 |
| Infections NEC | Infections - pathogen unspecified | 40 |
| Ectoparasitic infestations | Ectoparasitic disorders | 4 |
| Amoebic infections | Protozoal infectious disorders | 3 |
| **HLGT** | **SOC** | **Cases** |
| Infections - pathogen unspecified | Infections and infestations | 3926 |
| Infections - pathogen unspecified | Infections and infestations | 1737 |
| Infections - pathogen unspecified | Infections and infestations | 607 |
| Infections - pathogen unspecified | Infections and infestations | 585 |
| Infections - pathogen unspecified | Infections and infestations | 568 |
| Bacterial infectious disorders | Infections and infestations | 381 |
| Fungal infectious disorders | Infections and infestations | 266 |
| Infections - pathogen unspecified | Infections and infestations | 241 |
| Infections - pathogen unspecified | Infections and infestations | 210 |
| Infections - pathogen unspecified | Infections and infestations | 206 |
| Infections - pathogen unspecified | Infections and infestations | 175 |
| Infections - pathogen unspecified | Infections and infestations | 154 |
| Infections - pathogen unspecified | Infections and infestations | 152 |
| Infections - pathogen unspecified | Infections and infestations | 143 |
| Infections - pathogen unspecified | Infections and infestations | 142 |
| Bacterial infectious disorders | Infections and infestations | 139 |
| Viral infectious disorders | Infections and infestations | 132 |
| Fungal infectious disorders | Infections and infestations | 121 |
| Infections - pathogen unspecified | Infections and infestations | 115 |
| Infections - pathogen unspecified | Infections and infestations | 98 |
| Infections - pathogen unspecified | Infections and infestations | 92 |
| Infections - pathogen unspecified | Infections and infestations | 85 |
| Fungal infectious disorders | Infections and infestations | 85 |
| Infections - pathogen unspecified | Infections and infestations | 84 |
| Mycobacterial infectious disorders | Infections and infestations | 75 |
| Bacterial infectious disorders | Infections and infestations | 74 |
| Infections - pathogen unspecified | Infections and infestations | 73 |
| Infections - pathogen unspecified | Infections and infestations | 71 |
| Viral infectious disorders | Infections and infestations | 70 |
| Infections - pathogen unspecified | Infections and infestations | 63 |
| Infections - pathogen unspecified | Infections and infestations | 62 |
| Infections - pathogen unspecified | Infections and infestations | 61 |
| Infections - pathogen unspecified | Infections and infestations | 60 |
| Infections - pathogen unspecified | Infections and infestations | 57 |
| Bacterial infectious disorders | Infections and infestations | 56 |
| Infections - pathogen unspecified | Infections and infestations | 55 |
| Infections - pathogen unspecified | Infections and infestations | 54 |
| Viral infectious disorders | Infections and infestations | 51 |
| Bacterial infectious disorders | Infections and infestations | 48 |
| Fungal infectious disorders | Infections and infestations | 46 |
| Bacterial infectious disorders | Infections and infestations | 44 |
| Infections - pathogen unspecified | Infections and infestations | 44 |
| Viral infectious disorders | Infections and infestations | 43 |
| Fungal infectious disorders | Infections and infestations | 43 |
| Infections - pathogen unspecified | Infections and infestations | 40 |
| Ectoparasitic disorders | Infections and infestations | 4 |
| Protozoal infectious disorders | Infections and infestations | 3 |

**Supplementary Table 5.** Primary data for heatmap of ROR value.

| **PT** | **Pembrolizumab** | **Nivolumab** | **Cemlimab** | **Atezolizumab** | **Durvalumab** | **Avelumab** | **Ipilimumab** | **Tremelimumab** | **Nivo+Ipi** | **Pem+Ipo** | **Trem+Dur** | **Overall** |
| --- | --- | --- | --- | --- | --- | --- | --- | --- | --- | --- | --- | --- |
| Pneumonia | 1.27 | 1.91 | 2.05 | 2.43 | 2.74 | 1.15 | 1.80 | 0.92 | 1.59 | 0.98 | 1.43 | 1.81 |
| Sepsis | 1.60 | 2.68 | 4.00 | 3.75 | 2.05 | 2.19 | 3.18 | - | 3.25 | 1.55 | - | 2.54 |
| Encephalitis | 11.67 | 15.02 | 23.76 | 30.96 | 9.68 | 22.72 | 13.17 | - | 17.72 | - | - | 17.25 |
| Septic shock | 1.91 | 2.41 | 1.84 | 2.47 | 2.09 | 2.35 | 2.62 | 7.84 | 2.65 | 1.38 | - | 2.29 |
| Pneumonia aspiration | 3.77 | 4.30 | 5.06 | 3.05 | 4.01 | 2.68 | 2.65 | - | 2.61 | 2.37 | - | 3.86 |
| Pneumonia bacterial | 9.98 | 5.50 | 7.09 | 5.71 | 13.71 | 5.65 | 4.97 | - | 2.09 | - | - | 7.49 |
| Pneumocystis jirovecii pneumonia | 4.18 | 3.67 | 2.64 | 2.24 | 6.59 | - | 3.44 | - | 2.86 | 4.97 | - | 3.78 |
| Meningitis | 5.67 | 5.41 | 2.41 | 12.60 | 1.63 | 2.56 | 6.04 | - | 9.53 | - | - | 6.37 |
| Meningitis aseptic | 6.15 | 10.29 | 7.18 | 4.49 | 0.81 | 3.81 | 12.68 | - | 22.29 | 40.57 | - | 8.42 |
| Peritonitis | 1.07 | 1.44 | 1.38 | 2.16 | 0.78 | 2.20 | 2.42 | - | 1.36 | - | - | 1.50 |
| Device related infection | 1.53 | 1.65 | - | 1.82 | 0.77 | 6.35 | 1.11 | - | 0.92 | - | - | 1.57 |
| Conjunctivitis | 0.99 | 1.87 | 0.85 | 0.61 | 0.39 | 0.90 | 1.96 | - | 2.01 | 3.2 | - | 1.38 |
| Gastroenteritis | 1.12 | 1.79 | 1.09 | 1.75 | 2.72 | 2.32 | 2.95 | - | 2.36 | 8.23 | - | 1.75 |
| Bacteraemia | 1.09 | 2.13 | 5.38 | 4.09 | 2.13 | 1.43 | 1.61 | - | 2.24 | - | - | 2.03 |
| Pyelonephritis | 2.91 | 1.82 | 1.79 | 5.92 | 1.21 | 3.80 | 2.32 | - | 2.28 | - | - | 2.70 |
| Clostridium difficile colitis | 1.77 | 2.16 | 1.44 | 1.41 | 2.60 | 3.05 | 3.31 | - | 3.53 | - | - | 2.11 |
| COVID-19 pneumonia | 1.04 | 1.24 | 2.27 | 3.65 | 4.62 | 2.41 | 0.57 | - | 1.67 | - | - | 1.58 |
| Oral candidiasis | 1.20 | 2.13 | 1.26 | 1.57 | 0.85 | - | 1.26 | - | 2.35 | - | - | 1.61 |
| Urosepsis | 0.86 | 1.92 | 6.55 | 4.53 | 1.48 | 3.48 | 2.13 | - | 2.41 | - | - | 1.99 |
| Skin infection | 0.28 | 1.47 | 1.31 | 2.23 | 2.68 | 1.40 | 2.37 | - | 1.68 | - | - | 1.36 |
| Infectious pleural effusion | 9.48 | 7.10 | - | 11.41 | 31.25 | - | 9.81 | - | 5.76 | - | - | 10.16 |
| Appendicitis | 1.20 | 1.51 | 1.70 | 1.82 | 3.46 | 1.80 | 1.19 | - | 1.83 | - | - | 1.52 |
| Bronchopulmonary aspergillosis | 1.15 | 1.87 | - | 1.45 | 4.15 | - | 3.26 | - | 1.20 | - | - | 1.83 |
| Liver abscess | 3.24 | 4.34 | - | 7.34 | 13.14 | - | 0.48 | - | 0.95 | 18.12 | - | 4.37 |
| Pulmonary tuberculosis | 2.88 | 3.28 | 3.87 | 2.42 | 7.90 | - | 1.55 | - | 0.38 | - | - | 3.10 |
| Erysipelas | 1.23 | 2.61 | 2.92 | 2.87 | - | 6.20 | 3.80 | - | 1.15 | - | - | 2.29 |
| Lung abscess | 5.12 | 5.68 | - | 6.10 | 18.97 | - | 3.79 | - | 2.98 | - | - | 6.05 |
| Sialoadenitis | 6.46 | 9.79 | - | 8.11 | 7.69 | 12.01 | 11.40 | - | 5.57 | - | - | 9.01 |
| Coronavirus infection | 1.86 | 0.88 | - | 2.76 | 0.82 | 1.93 | 0.36 | - | 0.89 | - | - | 1.34 |
| Vascular device infection | 1.64 | 2.84 | 3.57 | 4.79 | 2.42 | - | - | - | 2.46 | - | - | 2.39 |
| Neutropenic sepsis | 0.73 | 0.85 | - | 4.65 | 0.47 | - | 2.08 | - | 1.43 | - | - | 1.36 |
| Myelitis | 3.79 | 6.94 | - | 9.60 | 7.26 | 22.73 | 11.85 | - | 11.64 | - | - | 7.21 |
| Endocarditis | 2.05 | 2.03 | 3.05 | 3.00 | - | - | 0.92 | - | 2.10 | - | - | 1.94 |
| Enterocolitis infectious | 1.18 | 9.23 | - | 24.50 | 38.53 | - | 13.56 | - | 15.01 | - | - | 10.91 |
| Relapsing fever | 3.45 | 87.80 | - | - | - | - | - | - | 4.78 | - | - | 37.79 |
| Pulmonary sepsis | 1.41 | 8.88 | - | 8.06 | 4.52 | - | 6.01 | - | 6.89 | - | - | 6.01 |
| Biliary tract infection | 0.86 | 14.29 | 24.40 | 31.33 | 79.62 | - | 4.88 | - | 2.39 | - | - | 15.56 |
| Cytomegalovirus enterocolitis | 10.82 | 9.53 | - | 7.67 | 9.70 | - | 30.72 | - | 8.45 | - | - | 12.60 |
| Escherichia urinary tract infection | 0.63 | 2.95 | - | 1.28 | 1.63 | - | 1.80 | - | 2.83 | - | - | 1.82 |
| Oesophageal candidiasis | 1.18 | 2.90 | - | 1.12 | 1.90 | - | 2.52 | - | 2.47 | - | - | 2.04 |
| Staphylococcal sepsis | 1.21 | 1.93 | 3.43 | 0.92 | - | - | 2.75 | - | 2.36 | - | - | 1.59 |
| Abdominal abscess | 1.24 | 1.07 | 3.51 | 3.13 | 0.79 | - | 3.51 | - | 2.07 | - | - | 1.63 |
| Bronchiolitis | 2.04 | 2.50 | - | 1.59 | 1.01 | 4.72 | 0.89 | - | 1.75 | - | - | 2.02 |
| Pneumonia fungal | 0.85 | 1.66 | 6.86 | 2.75 | 3.10 | - | 0.68 | - | 1.68 | - | - | 1.55 |

**Supplementary Table 6.** ROR value of the reporting top six ICI-related infectious adverse events at PT level for various treatment strategies.

|  | Pneumonia | | |  | Sepsis | | |  | Urinary tract infection | | |  | COVID-19 | | |  | Septic shock | | |  | Encephalitis | | |
| --- | --- | --- | --- | --- | --- | --- | --- | --- | --- | --- | --- | --- | --- | --- | --- | --- | --- | --- | --- | --- | --- | --- | --- |
|  | ROR | ROR_025_ | ROR_975_ |  | ROR | ROR_025_ | ROR_975_ |  | ROR | ROR_025_ | ROR_975_ |  | ROR | ROR_025_ | ROR_975_ |  | ROR | ROR_025_ | ROR_975_ |  | ROR | ROR_025_ | ROR_975_ |
| Pembrolizumab | 1.27 | 1.19 | 1.36 |  | 1.60 | 1.43 | 1.78 |  | 0.70 | 0.61 | 0.79 |  | 0.47 | 0.40 | 0.55 |  | 1.91 | 1.63 | 2.25 |  | 11.67 | 9.84 | 13.85 |
| Nivolumab | 1.91 | 1.82 | 2.00 |  | 2.68 | 2.49 | 2.88 |  | 0.90 | 0.82 | 0.99 |  | 0.55 | 0.48 | 0.61 |  | 2.41 | 2.13 | 2.72 |  | 15.02 | 13.18 | 17.12 |
| Cemlimab | 2.05 | 1.54 | 2.74 |  | 4.00 | 2.77 | 5.76 |  | 1.47 | 0.91 | 2.36 |  | 2.13 | 1.46 | 3.12 |  | 1.84 | 0.77 | 4.43 |  | 23.76 | 12.77 | 44.23 |
| Atezolizumab | 2.43 | 2.24 | 2.63 |  | 3.75 | 3.35 | 4.20 |  | 1.69 | 1.48 | 1.93 |  | 1.27 | 1.10 | 1.47 |  | 2.47 | 1.97 | 3.10 |  | 30.96 | 26.19 | 36.60 |
| Durvalumab | 2.74 | 2.43 | 3.08 |  | 2.05 | 1.61 | 2.61 |  | 0.58 | 0.41 | 0.84 |  | 1.05 | 0.81 | 1.36 |  | 2.09 | 1.41 | 3.09 |  | 9.68 | 6.09 | 15.38 |
| Avelumab | 1.15 | 0.78 | 1.71 |  | 2.19 | 1.32 | 3.63 |  | 0.64 | 0.31 | 1.35 |  | 0.25 | 0.08 | 0.78 |  | 2.35 | 1.06 | 5.24 |  | 22.72 | 11.80 | 43.72 |
| Ipilimumab | 1.80 | 1.63 | 1.98 |  | 3.18 | 2.79 | 3.62 |  | 0.85 | 0.70 | 1.04 |  | 0.29 | 0.21 | 0.40 |  | 2.62 | 2.08 | 3.31 |  | 13.17 | 10.09 | 17.18 |
| Tremelimumab | 0.92 | 0.13 | 6.56 |  | - | - | - |  | - | - | - |  | 3.36 | 0.83 | 13.54 |  | 7.84 | 1.10 | 55.96 |  | - | - | - |
| Pembrolizumab+Ipilimumab | 0.98 | 0.44 | 2.18 |  | 1.55 | 0.50 | 4.80 |  | 0.65 | 0.16 | 2.59 |  | - | - | - |  | 1.38 | 0.19 | 9.84 |  | - | - | - |
| Nivolumab+Ipilimumab | 1.59 | 1.43 | 1.76 |  | 3.25 | 2.86 | 3.69 |  | 0.95 | 0.79 | 1.14 |  | 0.59 | 0.48 | 0.74 |  | 2.65 | 2.10 | 3.33 |  | 17.72 | 14.10 | 22.27 |
| Tremelimumab+Durvalumab | 1.43 | 0.20 | 10.24 |  | - | - | - |  | - | - | - |  | 5.25 | 1.30 | 21.23 |  | - | - | - |  | - | - | - |

**Supplementary Table 7.** Tuberculosis infection associated with check point inhibitors reported in FAERS.

| SOC | HLGT | HLT | PT | Cases | ROR(95% CI) |
| --- | --- | --- | --- | --- | --- |
| Infections and infestations | Mycobacterial infectious disorders | Tuberculous infections | Tuberculosis | 77 | 0.96(0.77-1.20) |
| Infections and infestations | Mycobacterial infectious disorders | Tuberculous infections | Pulmonary tuberculosis | 75 | 3.10(2.47-3.90) |
| Infections and infestations | Mycobacterial infectious disorders | Tuberculous infections | Latent tuberculosis | 10 | 0.71(0.38-1.32) |
| Infections and infestations | Mycobacterial infectious disorders | Tuberculous infections | Lymph node tuberculosis | 6 | 1.31(0.58-2.92) |
| Infections and infestations | Mycobacterial infectious disorders | Tuberculous infections | Disseminated tuberculosis | 6 | 0.45(0.20-1.00) |
| Infections and infestations | Mycobacterial infectious disorders | Tuberculous infections | Intestinal tuberculosis | 2 | 1.75(0.43-7.06) |
| Infections and infestations | Mycobacterial infectious disorders | Tuberculous infections | Joint tuberculosis | 1 | 1.59(0.22-11.42) |

**Supplementary Table 8.** Herpes infection associated with check point inhibitors reported in FAERS.

| SOC | HLGT | HLT | PT | Cases | ROR(95% CI) |
| --- | --- | --- | --- | --- | --- |
| Infections and infestations | Viral infectious disorders | Herpes viral infections | Herpes zoster | 344 | 0.89(0.80-0.99) |
| Infections and infestations | Viral infectious disorders | Herpes viral infections | Oral herpes | 49 | 0.39(0.30-0.52) |
| Infections and infestations | Viral infectious disorders | Herpes viral infections | Herpes virus infection | 30 | 0.85(0.59-1.22) |
| Infections and infestations | Viral infectious disorders | Herpes viral infections | Herpes simplex | 22 | 0.74(0.49-1.13) |
| Infections and infestations | Viral infectious disorders | Herpes viral infections | Meningoencephalitis herpetic | 14 | 3.24(1.90-5.51) |
| Infections and infestations | Viral infectious disorders | Herpes viral infections | Ophthalmic herpes zoster | 10 | 0.80(0.43-1.49) |
| Infections and infestations | Viral infectious disorders | Herpes viral infections | Herpes ophthalmic | 8 | 0.91(0.45-1.82) |
| Infections and infestations | Viral infectious disorders | Herpes viral infections | Herpes zoster oticus | 6 | 1.96(0.87-4.39) |
| Infections and infestations | Viral infectious disorders | Herpes viral infections | Herpes simplex reactivation | 5 | 2.68(1.11-6.51) |
| Infections and infestations | Viral infectious disorders | Herpes viral infections | Herpes simplex encephalitis | 5 | 1.61(0.67-3.89) |
| Infections and infestations | Viral infectious disorders | Herpes viral infections | Herpes zoster reactivation | 4 | 2.52(0.93-6.78) |
| Infections and infestations | Viral infectious disorders | Herpes viral infections | Herpes oesophagitis | 3 | 2.45(0.78-7.69) |
| Infections and infestations | Viral infectious disorders | Herpes viral infections | Genital herpes | 3 | 0.29(0.09-0.91) |
| Infections and infestations | Viral infectious disorders | Herpes viral infections | Eczema herpeticum | 3 | 1.13(0.36-3.54) |
| Infections and infestations | Viral infectious disorders | Herpes viral infections | Ophthalmic herpes simplex | 3 | 0.75(0.24-2.33) |
| Infections and infestations | Viral infectious disorders | Herpes viral infections | Pneumonia herpes viral | 2 | 3.52(0.86-14.38) |
| Infections and infestations | Viral infectious disorders | Herpes viral infections | Herpes zoster disseminated | 2 | 0.45(0.11-1.81) |
| Infections and infestations | Viral infectious disorders | Herpes viral infections | Human herpesvirus 6 infection | 2 | 0.16(0.04-0.64) |
| Infections and infestations | Viral infectious disorders | Herpes viral infections | Genital herpes simplex | 2 | 1.41(0.35-5.68) |
| Infections and infestations | Viral infectious disorders | Herpes viral infections | Nasal herpes | 2 | 1.51(0.37-6.10) |
| Infections and infestations | Viral infectious disorders | Herpes viral infections | Herpes zoster meningitis | 2 | 1.72(0.43-6.95) |
| Infections and infestations | Viral infectious disorders | Herpes viral infections | Meningitis herpes | 2 | 2.42(0.60-9.83) |
| Infections and infestations | Viral infectious disorders | Herpes viral infections | Herpes zoster meningoencephalitis | 1 | 0.42(0.06-3.01) |
| Infections and infestations | Viral infectious disorders | Herpes viral infections | Herpes simplex pneumonia | 1 | 2.25(0.31-16.31) |
| Infections and infestations | Viral infectious disorders | Herpes viral infections | Herpes dermatitis | 1 | 0.85(0.12-6.06) |
| Infections and infestations | Viral infectious disorders | Herpes viral infections | Herpes zoster infection neurological | 1 | 0.98(0.14-7.01) |
| Infections and infestations | Viral infectious disorders | Herpes viral infections | Herpes pharyngitis | 1 | 2.62(0.36-19.03) |
| Infections and infestations | Viral infectious disorders | Herpes viral infections | Human herpesvirus 6 encephalitis | 1 | 0.66(0.09-4.73) |
| Infections and infestations | Viral infectious disorders | Herpes viral infections | Herpes zoster meningoradiculitis | 1 | 16.10(1.98-130.83) |
| Infections and infestations | Viral infectious disorders | Herpes viral infections | Herpes simplex viraemia | 1 | 1.37(0.19-9.87) |
| Infections and infestations | Viral infectious disorders | Herpes viral infections | Herpes zoster cutaneous disseminated | 1 | 0.57(0.08-4.08) |
| Infections and infestations | Viral infectious disorders | Herpes viral infections | Herpes simplex gastritis | 1 | 56.34(5.11-621.31) |

**Supplementary Table 9.** Outcomes of combination regimens associated iAEs

|  | Nivo+Ipi | Pembro+Ipi | Durva+Treme |
| --- | --- | --- | --- |
|  | 1895 | 29 | 5 |
| Life-Threatening | 264(13.93%) | 2(6.90%) | 1(20.00%) |
| Hospitalization | 1497(79.00%) | 17(58.62%) | 5(100.00%) |
| Death | 436(23.01%) | 3(10.34%) | 1(20.00%) |

**Supplementary Figure 1.** Forest plot of age-based disparities in infectious adverse events associated with immune checkpoint inhibitors. a: reports of target infectious adverse event occurrences in patients aged ≥65 years; b: reports of other adverse event occurrences in patients aged <65 years; c: reports of target infectious adverse event occurrences in patients aged <65 years; d: reports of other adverse event occurrences in patients aged ≥65 years.


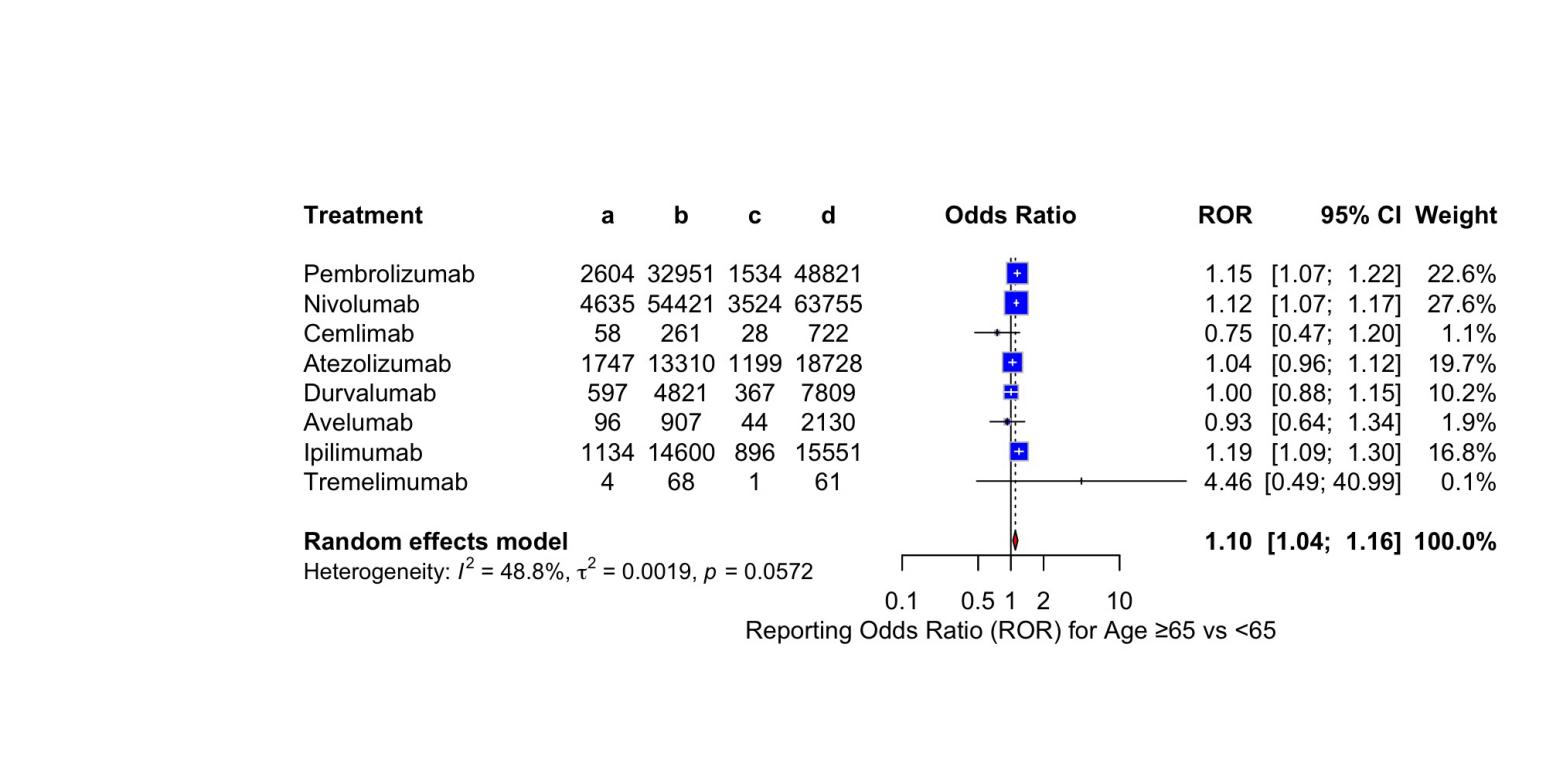

Supplement: Supplementary file 1 [file Table1.docx]
